# Unraveling the impact of operational parameters and environmental conditions on the quality of viable bacterial aerosols

**DOI:** 10.1093/pnasnexus/pgae473

**Published:** 2024-10-30

**Authors:** Mathura Thirugnanasampanthar, Lei Tian, Rod G Rhem, Danielle D Libera, Mellissa Gomez, Kyle Jackson, Alison E Fox-Robichaud, Myrna B Dolovich, Zeinab Hosseinidoust

**Affiliations:** Department of Chemical Engineering, McMaster University, 1280 Main Street West, Hamilton, ON, L8S 4L7, Canada; Department of Chemical Engineering, McMaster University, 1280 Main Street West, Hamilton, ON, L8S 4L7, Canada; Affiliate, Research Institute of St Joseph's Hospital and Firestone Institute for Respiratory Health, 50 Charlton Ave East, Hamilton, ON, L8N 4A6, Canada; Department of Biochemistry and Biomedical Sciences, McMaster University, 1280 Main Street West, Hamilton, ON, L8S 4K1, Canada; Department of Chemical Engineering, McMaster University, 1280 Main Street West, Hamilton, ON, L8S 4L7, Canada; Department of Chemical Engineering, McMaster University, 1280 Main Street West, Hamilton, ON, L8S 4L7, Canada; Farncombe Family Digestive Health Research Institute, McMaster University, Hamilton, ON, L8S 4K1, Canada; Department of Medicine, Faculty of Health Sciences, McMaster University, 1200 Main Street West, Hamilton, ON, L8N 3Z5, Canada; Centre of Excellence in Protective Equipment and Materials, McMaster University, 1280 Main Street West, Hamilton, ON, L8S 4L7, Canada; Farncombe Family Digestive Health Research Institute, McMaster University, Hamilton, ON, L8S 4K1, Canada; Department of Medicine, Faculty of Health Sciences, McMaster University, 1200 Main Street West, Hamilton, ON, L8N 3Z5, Canada; Centre of Excellence in Protective Equipment and Materials, McMaster University, 1280 Main Street West, Hamilton, ON, L8S 4L7, Canada; Department of Chemical Engineering, McMaster University, 1280 Main Street West, Hamilton, ON, L8S 4L7, Canada; Farncombe Family Digestive Health Research Institute, McMaster University, Hamilton, ON, L8S 4K1, Canada; Centre of Excellence in Protective Equipment and Materials, McMaster University, 1280 Main Street West, Hamilton, ON, L8S 4L7, Canada; School of Biomedical Engineering, McMaster University, 1280 Main Street West, Hamilton, ON, L8S 4L7, Canada; Michael DeGroote Institute for Infectious Disease Research, McMaster University, 1280 Main Street West, Hamilton, ON, L8S 4L8, Canada

## Abstract

Viable pathogen-laden droplets of consistent quality are essential for reliably assessing the protection offered by facemasks against airborne infections. We identified a significant gap in guidance within standardized tests for evaluating the filtration efficiencies of facemask materials using viable bacteria-laden aerosol droplets. An aerosol platform, built according to the American Society for Testing and Materials standard F2101-19, was used to validate and standardize facemask filtration test procedures. We utilized this platform to investigate the impact of varying five operating parameters, namely suspension media composition, relative humidity, pathogen concentration, and atomizer airflow and feed flow rates, on the aerosol quality of viable bacteria-laden aerosols. We achieved consistent generation of 1,700 to 3,000 viable bacteria-laden droplets sized between 2.7 and 3.3 µm under the following optimized test conditions: 1.5% w/v peptone water concentration, ≥80% relative humidity at 24 ± 2 °C, 1 × 10^5^ CFU/mL bacterial concentration, 1.5 L/min atomizer airflow rate, and 170 μL/min feed flow rate. We also explored the consequence of deviating from these optimized test parameters on viable bacteria-laden aerosol quality. These results highlight the importance of controlling these parameters when studying airborne transmission and control.

**Significance Statement**

Our research addresses a less explored aspect of air quality—the lack of reproducibility in standardized testing. We were part of a team that founded the Center of Excellence for Protective Equipment and Materials at McMaster University in the early days of the COVID-19 pandemic. Working closely with government regulators, government labs, and the industry sector, we realized that protective material, challenged with infectious aerosols according to standardized guidelines, received a different filtration efficiency rating in different labs worldwide and even in the same region. We found this observation alarming and a potential public health concern and thus spent 3+ years investigating the root cause of this lack of reproducibility.

## Introduction

Facemasks have been widely adopted by essential service workers, including healthcare professionals and the general public, to mitigate the transmission of airborne infections (1–3). The ability of facemasks to prevent inhalation of harmful particles and droplets can vary significantly depending on the filtration efficiency of the material used (4, 5). Materials with suboptimal filtration efficiency may endanger public health and impede effective disease control measures. Regulatory approval of mask products in many jurisdictions is contingent on the outcome of standardized filtration efficiency tests conducted per established regulatory guidelines, such as the American Society for Testing and Materials (ASTM) and the National Institute for Occupational Safety and Health (NIOSH) (6). Importantly, the manufacturing, sale, and commercialization of high-efficiency mask products cannot proceed without regulatory approval. Therefore, delays in obtaining reliable test results can directly impact the availability of mask products.

**Fig. 1.**
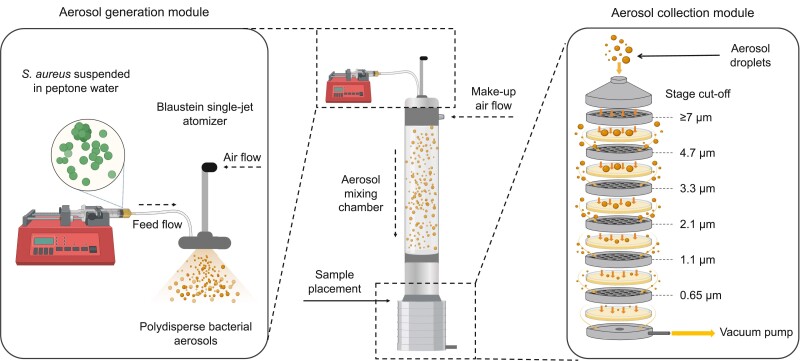
The illustration depicts the in-house experimental setup for BFE testing of facemask materials per ASTM standard F2101-19. Schematic of the BFE test workflow comprised of three main components: (left) the aerosol generation module, (center) a 60-cm-high glass aerosol mixing chamber, and (right) the aerosol collection module, all housed within a custom-built biological safety cabinet. Blaustein single-jet module, operated in atomizer mode, was used to generate polydisperse bacterial aerosol droplets. Glass Petri dishes prepared with 27 mL of semi-solid bacterial culture media were inserted into the impactor to collect size-fractioned droplets through impaction.

The shared experience of the pandemic coronavirus disease 2019 (COVID-19) brought attention to less scrutinized aspects of standard tests used to assess the quality of personal protective equipment, especially facemasks. The ASTM F2100-23 outlines a multitude of tests for evaluating medical facemask materials (7). More specifically, the ASTM F2101-19 provides the guidelines for conducting bacterial filtration efficiency (BFE) testing, which involves challenging mask materials with droplets containing *Staphylococcus aureus* (8). Due to the lack of a standardized viral aerosol testing method, procedures based on the ASTM F2101-19 standard have been widely adopted to assess the filtration performance of facemasks using viable bacteria-laden aerosols (9, 10). The F2101-19 standard outlines procedures for the generation, collection, and enumeration of these infectious aerosols (Fig. 1), mandating that test materials be challenged with 1,700 to 3,000 viable bacteria-laden droplets with a mean aerodynamic size of 2.7 to 3.3 μm, delivered over 2 min (8). However, the F2101-19 standard neglects to specify nearly half of the hardware specifications and operating conditions required to generate bacterial aerosols within the mandated range necessary for conducting filtration tests (Table 1). The operating conditions highlighted in Table 1 can take significant time to optimize. This delay can lead to lost time and capital, inconsistent outcomes between labs, and impede third-party verification of test results.

**Table 1.** Operating variables in bacterial aerosol generation, collection, and mask challenge.

	Variables	Values specified in ASTM F2101-19	Test values
Aerosol generation	Peptone water concentration (% w/v)^a^	—	**1.5**, 3.0, 6.0
	Temperature (°C)^a^	—	**24 ± 2**
	Relative humidity (%)^a^	—	0–20, 40–60, **80–100**
	*S. aureus* concentration (CFU/mL)^a^	∼1 × 10^5^	1 × 10^4^, **1** **×** **10^5^,** 1 × 10^6^
	Atomizer airflow rate (L/min)^a^	—	0.5, **1.5**, 2.5
	Atomizer feed flow rate (µL/min)^a^	—	100, **170**, 240
	Aerosol generation module	—	Blaustein atomizer single-jet module
	Atomizer feed flow duration (min)	1	1
	Atomizer airflow duration (min)^b^	2	4
	Aerosol chamber dimensions (cm)^c^	8 × 60	8 × 60
	Total counts (CFU)^d^	2,350 **±** 650	2,350 **±** 650
	Mean size (µm)	3.0 **±** 0.3	3.0 **±** 0.3
Aerosol collection	Glass Petri dish dimensions (mm)	15 × 100	15 × 100
	TSA volume (mL)^d^	—	27
	Impactor airflow rate (L/min)	28.3	28.3
	Impactor airflow duration (min)^b^	2	4
	Aerosol collection module	Six-stage viable cascade impactor	Six-stage viable cascade impactor
Aerosol enumeration	Incubation temperature (°C)	37 **±** 2	37 **±** 2
	Incubation humidity (%)	—	40–60
	Incubation time (h)^e^	48 **±** 4	24 **±** 4
Sample preparation	Mask conditioning temperature (°C)	21 **±** 5	21 **±** 5
	Mask conditioning humidity (%)	85 **±** 5	85 **±** 5
	Mask conditioning duration (h)	≥4	≥4
	Mask challenge area (cm^2^)	—	49
	Airflow velocity through mask (cm/s)	—	9.6

^a^Bolded test values indicate ambiguous or unspecified variables involved in bacterial aerosol generation. Test values of four of the five variables remained constant while altering a single variable.

^b^Atomizer and impactor air flow was maintained for 2 min prior to aerosol generation to allow humidity and temperature within the system to stabilize.

^c^The glass chamber functions as a baffle for the Blaustein atomizer which enables it to be operated as a nebulizer within the platform (11).

^d^Collection medium volume and co-incidence-corrected table values are based on publications by Macher (12) and Andersen (13).

^e^ATCC 6538 product handling sheet specifies a shorter incubation period of 24 h (14).

We developed and optimized an in-house custom aerosol platform per the ASTM F2101-19 specifications to address the identified knowledge gap (8). Utilizing the platform, the variation in the quality of generated bacterial aerosols in response to the manipulation of five key unspecified parameters, highlighted in Table 1, was explored. Experimental results were analyzed and interpreted according to established theories in aerosol transport mechanics, elucidating the conditions necessary for generating bacterial aerosols of consistent quality.

## Materials and methods

### Solution preparation

The saline solution contained 9 g of sodium chloride (Fisher Scientific S271-500) in 1 L of deionized water. Tryptic soy broth (TSB) medium contained 30 g of TSB (BD B211825) in 1 L of deionized water. Tryptic soy agar (TSA) medium contained 30 g of TSB (BD B211825) and 15 g of agar (Fisher BioReagents BP1423500) in 1 L of deionized water. Peptone water solutions contained varying concentrations of 15, 30, and 60 g/L of peptone water (Thermo Scientific CM0009) in ultrapure water (MilliporeSigma SYNSVHFUS). A precision balance (Mettler Toledo ME203E) was used to weigh out the dry reagents; all solutions were sterilized by autoclaving at 121 °C for 20 min (Yamato Steam Sterilizer SM301).

### Bacterial aerosol generation, collection, and detection

As depicted in Fig. 1, three components comprise the aerosol exposure platform: Blaustein atomizer single-jet model (CH Technologies ARGBLM2), a 60-cm-high and 8-cm-wide glass aerosol mixing chamber, and a six-stage viable cascade impactor (Tisch Environmental TE-10-800). A PushSensor thermometer–hygrometer (HT.w 16794383), placed inside the biological safety cabinet with the aerosol platform, was used to monitor ambient temperature and humidity fluctuations. Atomization of bacterial suspensions containing *S. aureus* (ATCC 6538) generates viable bacteria-laden aerosol droplets. Compressed air, filtered through an inline HEPA capsule filter (TSI 1602051), was delivered to the atomizer at a controlled flow rate (0.5, 1.5, or 2.5 L/min). A volumetric airflow rate of 28.3 L/min was maintained within the system using a downstream vacuum pump (GAST 0823-101Q-G608NEX) and monitored using a downstream flow meter (OMEGA FMA-A2317). Glass Petri dishes (Pyrex 3160-100) prepared with 27 mL of TSA were placed beneath each impactor stage to collect aerosols through impaction. The airflow through the system was maintained for 2 min to stabilize the humidity and temperature before aerosol generation. The 4-min experimental runtime consisted of 2 min of airflow before aerosol generation, 1 min of aerosol generation, and 1 min of airflow post-aerosol generation. Airflow continued for an additional minute after the liquid feed flow stopped to allow all generated droplets to reach the impactor. Aerosol collection plates were incubated at 37 °C for 24 ± 4 h to allow bacterial colony formation (14). Supplementary Material Sections S1–S3 (Tables S1 and S2; Figs. S1 and S2) provide additional details on platform operation, test value selection, and loss quantification.

### Determining the mean size and total count of viable bacteria-laden droplets

Bacterial colonies on collection plates were manually counted and converted into co-incidence-corrected whole-number values using a positive-hole correction factor to account for the co-incidence error (13). The mean aerodynamic diameter of viable bacteria-laden droplets, ${\bar{D}}_{\mathrm{ae}}$ (µm), was calculated according to Eq. 1, where *d*_50_ specifies the stage cutoff diameter, *i* specifies the impactor stage number, *N*_c_ is the co-incidence-corrected stage droplet counts, and *N*_T_ specifies the co-incidence-corrected count for viable bacteria-laden droplets across all six stages (15, 16). The total count of viable bacteria-laden droplets for the six stages of the impactor, *N*_T_, was calculated according to Eq. 2, where *N*_c_ is the co-incidence-corrected stage count and *i* specifies the impactor stage number (13). For additional details, including information on loss quantification within the system, refer to Supplementary Material Sections S1 and S2.


(1) $${\bar{D}}_{\mathrm{ae}}=\frac{\sum_{i=1}^{6}\;d_{50,i}\times N_{c,i}}{N_{T}}$$

(2) $$N_{T}=\overset{6}{\underset{i=1}{\sum }}\;N_{c,i}\;$$

### Determining the BFE and airflow resistance of test materials

BFE trials adhered to the ASTM standard F2101-19 specifications (8). The total count and mean size of viable bacteria-laden droplets composing the aerosol challenge were determined using two positive control runs conducted before and after test material challenges. The negative control run, performed at the end of the experimental period, was used to verify the sterility of the system. Microbial growth was absent on all negative control plates.

Test materials for BFE determination included commercial respirators and masks: Kimberly-Clark, Kimtech N95 Pouch Respiratory (NIOSH 53358), 3-Ply Face Masks (Amazon B08R5P19BX), and Levi's 100% cotton bandanas (Amazon B09697R47T). The test material, placed between the inlet cone and the first stage of the impactor, covers an area of 49 cm^2^ with the outer surface oriented toward the aerosol challenge to simulate mask protection of the wearer against infectious aerosols (17). The 49-cm^2^ area of the material experienced an airflow velocity of 9.6 cm/s, with droplets delivered at a volume flow rate of 28.3 L/min. The droplets that penetrate the test material, collected within the impactor according to aerodynamic size, were used to calculate the filtration efficiency of the material.

BFE expresses the fraction of viable droplets captured by the material relative to the number of viable droplets encountered by the material. BFE values were calculated according to Eq. 3, where *N*_AVG_ is the number of viable bacteria-laden droplets encountered by the material (based on the average of two positive control runs) and *N*_M_ is the number of droplets penetrating the test material. For additional details, refer to Supplementary Material Sections S1 and S7.


(3) $$\mathrm{BFE}\;(\text{\%})=\frac{\left(N_{\mathrm{AVG}}-N_{M}\right)}{N_{\mathrm{AVG}}}\times 100\text{\%}\;$$

The differential pressure (DP) test assesses resistance to airflow across the test material. DP tests were performed according to standard specifications, subjecting test materials to 27 cm/s airflow velocity, while the BFE testing subjects the material to 9.6 cm/s airflow velocity (7, 8, 18 ). For additional details regarding DP testing, refer to Supplementary Material Section S2.

### Electron microscopy of test materials

Scanning electron microscopy (SEM) was used to visualize the microstructural features of mask materials. Samples, coated with a 10-nm layer of gold (Edwards High Vacuum Coater S150B), were imaged (Tescan VEGA-II LSU) using 10 kV accelerating voltage, 60× magnification, 16.66 mm working distance, and 3.61 mm view field.

### Statistical analysis

Group means were analyzed using ordinary one-way ANOVA followed by Tukey's multiple comparisons test in GraphPad PRISM 9. The threshold for significance was *P*-value < 0.05. Group means from five independent aerosol trials were used to evaluate the effect of changing test values on total counts and mean size of viable bacteria-laden droplets.

## Results and discussion

### Effect of peptone water concentration on viable bacteria-laden aerosol properties

**Fig. 2.**
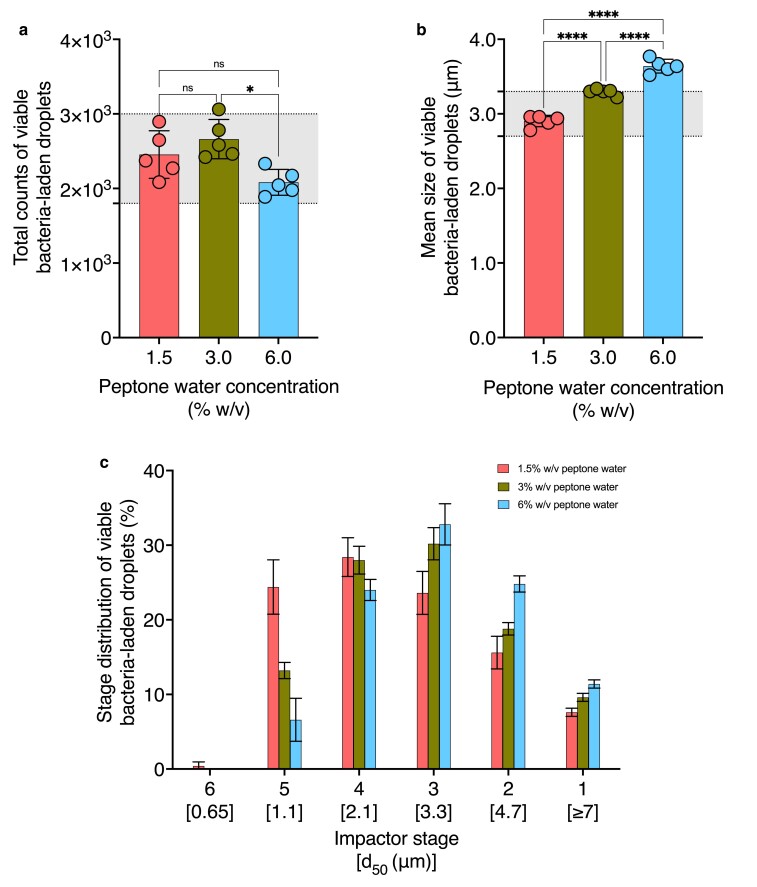
Effect of peptone water concentration on the mean size, total count, and stage distribution of viable bacteria-laden droplets. The impact of varying peptone water concentrations on a) mean size, b) total count, and c) stage distribution of viable bacteria-laden droplets is shown (*n* = 5). All other operating variables for aerosol generation were kept constant according to Table S3 as peptone water concentration was varied, while the remaining parameters were adjusted as specified in Table 1. Regions shaded in gray indicate the ASTM standard F2101-19-specified range for a) mean size and b) total count of viable bacteria-laden droplets. Asterisks indicate statistically significant differences, defined as *P-value < 0.05, **P-value <0.01, ***P-value <0.001, ****P-value <0.0001.

To assess the impact of suspension media concentration on aerosol properties, we quantified the mean size (Fig. 2a), total counts (Fig. 2b), and stage distribution (Fig. 2c) of viable bacteria-laden droplets generated from 1.5, 3, and 6% w/v peptone water suspensions. Without guidance from the standard and convention in applied microbiology, we selected peptone water concentrations (1.5, 3, and 6% w/v) to reflect sputum solids concentrations in healthy, moderate, and severe muco-obstructive airway disease states (19). The solid fraction of human airway mucus is a mixture of mucin, salts, globular proteins, lipids, DNA, and cellular debris (20, 21). The complex composition of mucus is challenging to simulate. However, the prepared peptone water solutions contain similar concentrations of solids (proteins and salts) to reflect that of airway mucus in health and disease states (19, 22). Table S3 provides the operating values held constant during these trials. Tables S4–S6 provide stage-specific viable bacteria-laden droplet count data for the three peptone water concentrations.

The mean size of viable bacteria-laden droplets increased from 2.90 ± 0.08 to 3.29 ± 0.04 and 3.64 ± 0.09 μm with increases in peptone water concentration (Fig. 2a). Total droplet counts increased from 2,455 ± 320 to 2,662 ± 263 before decreasing to 2,083 ± 174 for 1.5, 3.0, and 6.0% w/v peptone water concentrations, respectively (Fig. 2b). Figure 2c shows the stage distribution percentage of viable bacteria-laden droplets under varied peptone water concentrations. The collection of viable bacteria-laden droplets generated from 1.5% w/v peptone water solutions is left-skewed, with the highest fraction of droplets collecting below stage four with a 2.1-μm cutoff diameter (Fig. 2c). Meanwhile, the highest percentages of viable bacteria-laden droplets generated from 3 to 6% w/v peptone water solutions were collected below the third impactor stage with a 3.3-μm cutoff diameter (Fig. 2c). Only bacterial suspensions prepared in 1.5 and 3% w/v peptone water concentrations produced aerosol droplets within the ASTM-specified size and count range, as indicated by the shaded regions in Fig. 2a and b, respectively. Notably, 3% w/v peptone water concentration resulted in bacteria-laden droplets at the upper limit of the acceptable size range, while 6% w/v peptone water concentration produced droplets greater than the specified size range.

### Effect of relative humidity on viable bacteria-laden aerosol properties

**Fig. 3.**
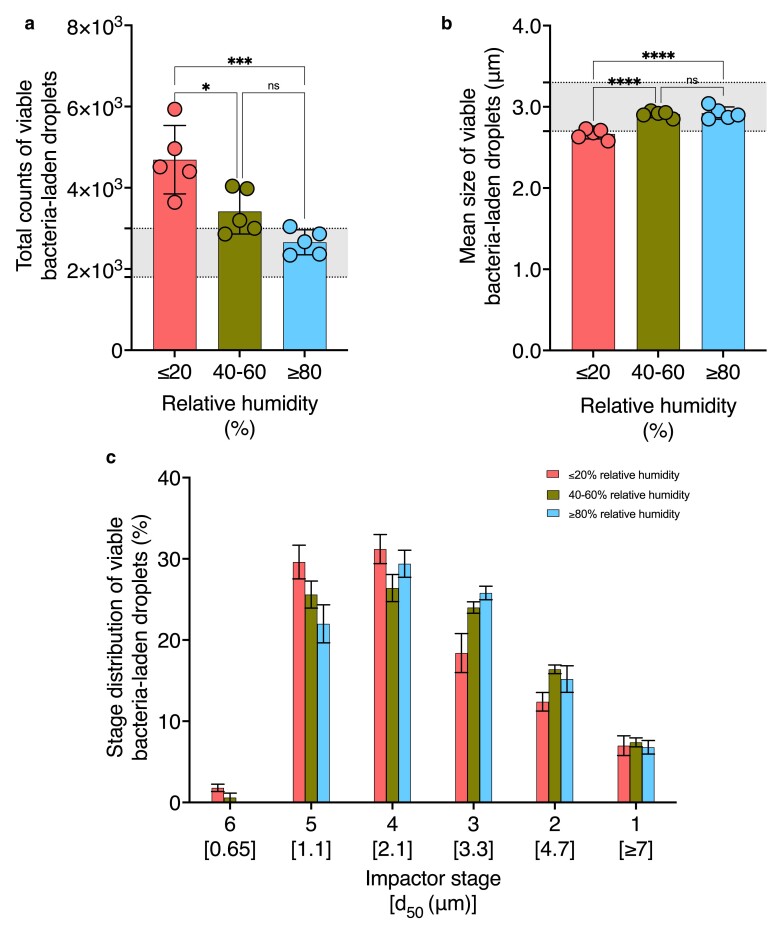
Effect of relative humidity on the mean size, total count, and stage distribution of viable bacteria-laden droplets. The impact of varying the relative humidity on a) mean size, b) total count, and c) stage distribution of collected viable bacteria-laden droplets is shown (*n* = 5). All other operating variables for aerosol generation were kept constant according to Table S7 as the relative humidity was varied, while the remaining parameters were adjusted as specified in Table 1. Regions shaded in gray indicate ASTM standard F2101-19-specified range for a) mean size and b) total count of viable bacteria-laden droplets. Asterisks indicate statistically significant differences, defined as *P-value < 0.05, **P-value <0.01, ***P-value <0.001, ****P-value <0.0001.

To examine the impact of RH on aerosol properties, we measured the mean size (Fig. 3a), total counts (Fig. 3b), and stage distribution (Fig. 3c) of viable bacteria-laden droplets under conditions of RH ≤20%, 40–60%, and ≥80%. Table S7 provides the operating values held constant during these trials. Tables S8–S10 provide stage-specific viable bacteria-laden droplet count data collected under varied RH conditions. The mean size of viable bacteria-laden droplets increased from 2.67 ± 0.06 to 2.91 ± 0.04 and 2.92 ± 0.08 μm with increasing RH (Fig. 3a). Total counts of viable bacteria-laden droplets decreased from 4,691 ± 842 to 3,416 ± 556 and 2,658 ± 307 under increasing RH (Fig. 3b). The highest percentages of viable bacteria-laden droplets exposed to ≤20% RH conditions were collected below the fourth and fifth impactor stages with cutoff diameters of 2.1 and 1.1 μm, respectively (Fig. 3c). Under high ≥80% RH conditions, the highest percentages of viable bacteria-laden droplets were collected below the third and fourth stages with cutoff diameters of 3.3 and 2.1 μm, respectively (Fig. 3c).

Only the mean size and total counts of viable bacteria-laden droplets collected under the ≥80% RH condition fell within the ASTM-specified range, as indicated by the shaded regions in Fig. 3a and b, respectively. This finding highlights a pitfall of the ASTM F2101-19 standard; different geographical locations or weather conditions may alter the quality of the aerosol. The range of RH that produces the required quality of aerosols is not naturally achievable in many parts of the world or all year round. A humidity-controlled chamber or laboratory may be required to produce bacterial aerosols of consistent quality.

The noticeable increase in the mean size of viable bacteria-laden droplets, observed under increasing peptone water concentration (Fig. 2a) or increasing RH (Fig. 3a), can be explained by the factors affecting the equilibrium between the droplet phase and gas phase water activity. The composition of peptone water, the bacterial suspension medium specified by the ASTM, is a 2:1 mixture of peptone and sodium chloride, a hygroscopic salt (8). Increased concentration of hygroscopic solutes decreases droplet phase water activity (23), promoting moisture absorption and resulting in larger equilibrated droplet sizes (Fig. 2a). Meanwhile, increased RH within the aerosol mixing chamber reflects a higher gas phase water activity, driving the phase equilibrium toward the swelling of droplets containing hygroscopic solutes (Fig. 3a). The interplay between solute concentration, hygroscopicity, and humidity contributes to the observed trends in bacteria-laden droplet size (Figs. 2a and 3a). Equation 4 describes this interplay, where $R_{\mathrm{eq}}$ (µm) is the equilibrium radius of a droplet, $R_{0}$ (µm) is the starting radius of the droplet, $C_{s}$ (g/mL) is the peptone water concentration, and RH is the relative humidity (24). This equation illustrates why the equilibrium radius of the droplet will increase with the increase in solute concentration or RH when the remaining parameter is held constant.


(4) $$R_{\mathrm{eq}}=R_{0}{\left(\frac{C_{s}}{1-\mathrm{RH}}\right)}^{\frac{1}{3}}$$

Unlike the trend observed for the mean size, a significant decrease in total counts of viable bacteria-laden droplets occurred with increasing peptone water concentration (Fig. 2b) or RH (Fig. 3d). This decline in total counts may result from droplet loss along the walls of the aerosol mixing chamber, primarily due to gravitational sedimentation and inertial impaction. Gravitational sedimentation refers to the settling of droplets (1 to 8 μm) under the force of gravity; inertial impaction refers to the deposition of droplets (>5 μm) due to abrupt changes in the carrier flow velocity (25). The terminal settling velocity characterizes gravitational sedimentation, signifying the faster settling of larger droplets (26). Using Eq. 5, the terminal settling velocity $V_{s}$ (m/s) of droplets can be calculated, where $\rho_{d}$ (kg/m^3^) is the droplet density, $D_{d}$ (m) is the droplet diameter, *g* (m/s^2^) is the gravitational acceleration, and *μ* (kg/m•s) is the dynamic viscosity of the carrier medium (25, 26). Specifically, Eq. 6 shows droplets with a larger diameter will experience a higher settling velocity.


(5) $$V_{s}=\frac{{\rho_{d}D_{d}}^{2}}{18\mu }g$$

The Stokes number governs inertial impaction, where a higher value represents an increased likelihood of deposition for larger droplets (inertial impaction at bends) and narrow regions of the chamber (inertial impaction due to flow constriction) (26). The dimensionless Stokes number Stk can be calculated according to Eq. 6, where $\rho_{d}$ (kg/m^3^) is the droplet density, $D_{d}$ (m) is the droplet diameter, *u* (m/s) is the mean velocity of the carrier medium, *μ* (kg/m•s) is the dynamic viscosity of the carrier medium, and *d* (m) is the diameter of the tube (25, 26). Equation 6 indicates that if all other parameters are kept constant, droplets with a larger diameter will have a higher Stokes number and, thus, a higher likelihood of deposition (25, 26). Equations 5 and 6 explain why increased wall loss may lead to decreased droplet counts as droplet size increases (Figs. 2 and 3).


(6) $$\mathrm{Stk}=\frac{\rho_{d}{D_{d}}^{2}u}{18\mu d}$$

Both deposition mechanisms, namely gravitational sedimentation and inertial impaction, can impact relaxation time, the time required for aerosol droplets to adapt to changes in the velocity of the carrier medium (27). Droplet relaxation time *τ* (s), calculated according to Eq. 7, where $D_{\mathrm{ae}}$ (m) is the droplet aerodynamic diameter, $\rho_{o}$ (kg/m^3^) is the density of water, $C_{C}$ is the Cunningham slip correction factor, and *μ* (kg/m•s) is the gas viscosity (27, 28)


(7) $$\tau =\frac{{D_{\mathrm{ae}}}^{2}\rho_{o}C_{c}}{18\mu }$$

As shown in Eq. 7, with increases in aerodynamic size, droplet relaxation time also increases, contributing to the loss of larger droplets that require more time to adjust to the changing trajectories of the gaseous carrier medium. The tendency of larger droplets to experience significant deposition within the system explains the decrease in total counts of viable bacteria-laden droplets with increasing peptone water concentration (Fig. 2b) or RH (Fig. 3b). The interplay between gravitational sedimentation, inertial impaction, relaxation time, and droplet size underscores the dynamic behavior of aerosol droplets within the platform and substantiates the observed trends in droplet counts.

### Effect of bacterial concentration on viable bacteria-laden aerosol properties

**Fig. 4.**
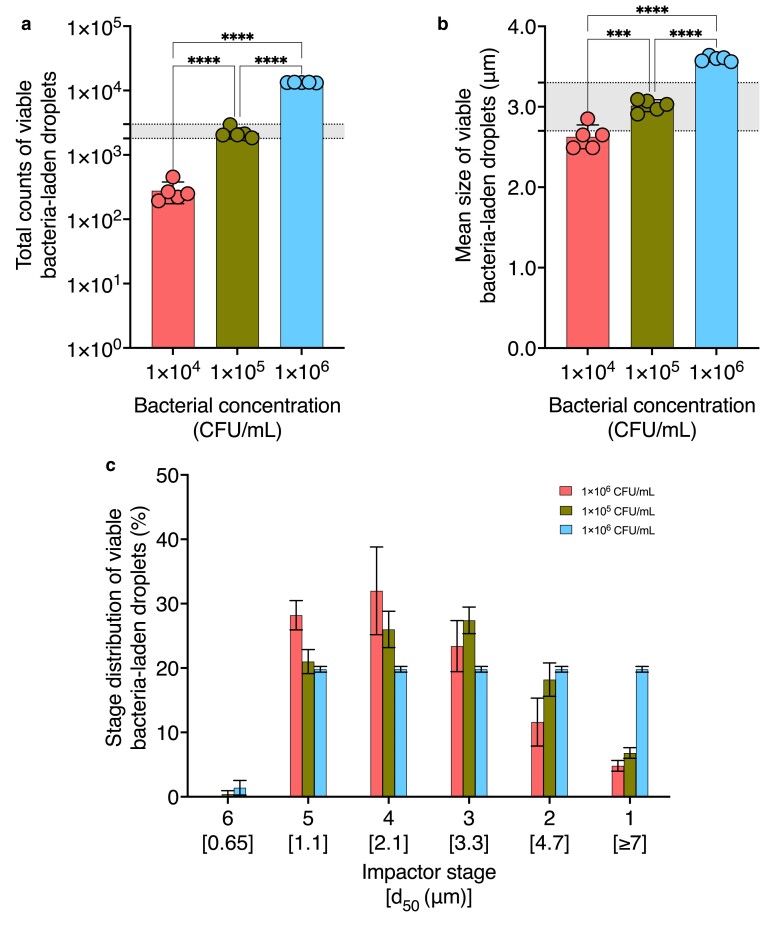
Effect of bacterial concentration on the mean size, total counts, and stage distribution of viable bacteria-laden droplets. The impact of varying bacterial concentrations on a) mean size, b) total count, and c) stage distribution of viable bacteria-laden droplets is shown (*n* = 5). All other operating variables for aerosol generation were kept constant according to Table S11 as bacterial concentration was varied. The remaining parameters were adjusted as specified in Table 1. Regions shaded in gray indicate the ASTM standard F2101-19-specified range for a) mean size and b) total count of viable bacteria-laden droplets. Asterisks indicate statistically significant differences, defined as *P-value < 0.05, **P-value <0.01, ***P-value <0.001, ****P-value <0.0001.

To examine the impact of pathogen concentration on viable aerosol properties, we quantified the mean size (Fig. 4a), total count (Fig. 4b), and stage distribution (Fig. 4c) of viable bacteria-laden droplets generated from feed suspensions with concentrations of 1 × 10^4^, 1 × 10^5^, and 1 × 10^6^ CFU/mL. The standard discusses a 5 × 10^5^ CFU/mL bacterial concentration and diluting as needed to reach the specified size and count range (8). Based on this guideline, we selected 1 × 10^5^ CFU/mL as a midpoint value and included concentrations one logarithmic order higher and lower, covering a relevant concentration range. Table S11 provides the operating values held constant during these trials. Tables S12–S14 provide stage-specific viable bacteria-laden droplet count data using varied bacterial feed suspensions.

The mean size of viable bacteria-laden droplets exhibited an increase from 2.63 ± 0.15 and 3.01 ± 0.07 to 3.60 ± 0.03 μm, corresponding to a logarithmic increase in bacterial concentration (Fig. 4a). Correspondingly, total counts varied from 277 ± 103 and 2,197 ± 435 to 13,339 ± 133 for 1 × 10^4^, 1 × 10^5^, and 1 × 10^6^ CFU/mL feed concentrations, respectively (Fig. 4b). Figure 4c shows the stage distribution of viable bacteria-laden droplets generated from three bacterial feed concentrations. A large fraction of viable bacteria-laden droplets generated from 1 × 10^4^ CFU/mL feed suspensions were collected below stages 4 and 5 with lower cutoff sizes (Fig. 4c). Meanwhile, droplets generated from 1 × 10^5^ CFU/mL feed suspensions were primarily collected below stages 3 and 4 with larger cutoff sizes (Fig. 4c). A feed concentration of 1 × 10^5^ CFU/mL was the only condition that resulted in droplets within the size and count range specified by the ASTM, as indicated by the shaded regions in Fig. 4a and b, respectively.

We atomized 0.17 mL of 1 × 10^4^, 1 × 10^5^ and 1 × 10^6^ CFU/mL bacterial feed suspensions, resulting in 1,700, 17,000, and 170,000 viable bacterial cells in the volume of atomized feed, respectively. We collected 277 ± 103 viable bacteria-laden droplets from the atomization of 0.17 mL of 1 × 10^4^ CFU/mL (*n* = 5). The atomized feed contained ∼1,700 bacterial cells. If all bacterial cells remained viable and distributed to a single droplet, 277 viable bacteria-laden droplets would indicate that 16% of the generated droplets reach the impactor and are collected.

Additionally, we collected 2,197 ± 435 viable bacteria-laden droplets from the atomization of 0.17 mL of 1 × 10^5^ CFU/mL (*n* = 5). The atomized feed contained ∼17,000 bacterial cells. Assuming each bacterial cell remained viable and distributed to a single droplet, 2,200 droplets indicate that 13% of the generated droplets reach the impactor and are collected. Lastly, we collected 13,339 ± 133 viable bacteria-laden droplets from the atomization of 0.17 mL of 1 × 10^6^ CFU/mL. The atomized volume contained ∼170,000 bacterial cells. If the same assumption is applied, 13,339 droplets represent 7.8% of the generated droplets.

The total viable bacteria-laden droplet counts representing <16% fraction would suggest the presence of more than one viable bacterial cell within collected droplets. Based on the above estimations, the number of bacterial cells per droplet is likely increasing from 1 cell per droplet in 1 × 10^4^ CFU/mL condition to >1 cell per droplet for 1 × 10^5^ CFU/mL condition and ∼2 cells per droplet for 1 × 10^6^ CFU/mL condition. However, a fraction of the droplets in the 1 × 10^5^ and 1 × 10^6^ CFU/mL conditions likely contain a single viable bacterial cell, while other droplets contain 2–4 bacterial cells.

The increased mean size of viable bacteria-laden droplets with increasing bacterial concentration might arise from the diffusional mixing rate of bacteria within (29). The diffusivity constant of a particle $D_{i}$ (cm^2^/s) can be calculated using Eq. 8, where $k_{B}$ (dyn•cm/K) is the Boltzmann's constant, *T* (*K*) is the temperature in Kelvin, $u_{i}$ (Pa•s) is the viscosity of the solvent medium, and $r_{i}$ (cm) is the radius of the diffusing particle (29).


(8) $$D_{i}=\frac{k_{B}T}{6\pi u_{i}r_{i}}\;$$

According to the Stokes–Einstein equation, an inverse relationship exists between the diffusional mixing rate of a particle within a suspended droplet (which is proportional to the diffusivity, $D_{i}$) and the radius of the diffusing particle ($r_{i}$). Staphylococci, with an approximate size of 1 µm, will exhibit much slower rates of diffusional mixing within droplets than smaller water molecules and dissolved solutes (30). As the droplet surface recedes, bacterial cells become concentrated near the droplet surface, while water molecules (assumed to be 0.2 nm in size) readily diffuse inwards (31). Consequently, bacterial cells may act as a barrier, impeding evaporative moisture loss by limiting the droplet surface area in contact with the environment (32, 33). Thus, the observed increase in the mean size of viable bacteria-laden droplets with bacterial concentration may be attributed to the moisture retention within droplets containing a higher bacterial load (Fig. 4a).

In addition, the kinetic coagulation of aerosol droplets is another mechanism that may contribute to the increase in the mean size of viable bacteria-laden droplets. Coagulation is a process by which droplets collide to produce a larger droplet, and kinematic coagulation describes a process governed by forces in addition to Brownian diffusion (as is the case within our system) (34). It is likely that both the kinematic coagulation process and higher bacterial load within droplets lead to the observed increase in the mean size of viable bacteria-laden droplets generated from the more concentrated feed suspensions (Fig. 4).

The increase in viable bacteria-laden droplet count with logarithmic increases in bacterial concentration (Fig. 4b) can be explained, in part, by an increasing number of bacteria-containing droplets, leading to a higher count of droplets detected through culture methods. Fernandez et al. (32) investigated the correlation between the bacterial feed concentration and the number of bacterial cells contained within generated droplets . Equation 9 shows the probability distribution function representing the likelihood of occurrence of a certain number of bacterial cells within aerosol droplets, where *k* is the number of cells contained in a droplet and *λ* is the Poisson distribution coefficient (average of cells per droplet for the bacterial feed concentration loaded into the droplet generator) (32).


(9) $$\mathrm{PDF}\;(\kappa )=\frac{\lambda^{-\kappa }}{\kappa !}e^{-\lambda }$$

Equation 9 represents a log-linear model supported by empirical data, illustrating that an increase in bacterial feed concentration raises the probability of droplets containing more than one bacterial cell. The research conducted by Liang et al. (33) complements this equation, presenting evidence that bacterial viability within aerosol droplets is positively affected by an increased density of bacterial cells. The authors propose that a higher number of cells aid in mitigating the osmotic stress experienced by bacteria within the aerosol droplet, ultimately contributing to enhanced bacterial viability, termed the population effect (33). This effect could lead to more droplets harboring more than one bacterial cell and a corresponding increase in total counts of viable bacteria-laden droplets (Fig. 4).

### Effect of atomizer airflow rate on viable bacteria-laden aerosol properties

**Fig. 5.**
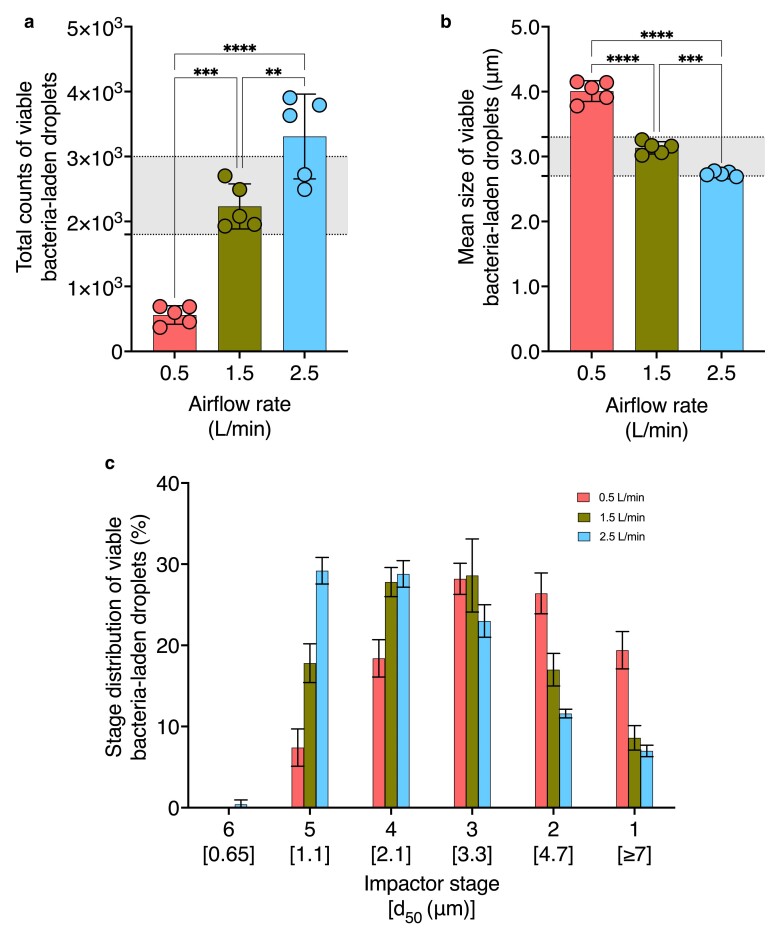
Effect of atomizer airflow rate on mean size, total counts, and stage distribution of viable bacteria-laden droplets. The impact of varying atomizer airflow rates on a) mean size, b) total counts, and c) stage distribution of viable bacteria-laden droplets is shown (*n* = 5). All other operating variables for aerosol generation were kept constant according to Table S15 as the atomizer airflow rate was varied. The remaining parameters were adjusted as specified in Table 1. Regions shaded in gray indicate ASTM standard F2101-19-specified range for a) mean size and b) total count of viable bacteria-laden droplets. Asterisks indicate statistically significant differences, defined as *P-value < 0.05, **P-value <0.01, ***P-value <0.001, ****P-value <0.0001.

Given the absence of a specific aerosol generation module in the ASTM standard, we opted for the Blaustein single-jet module operated in atomizer mode to generate bacteria-laden droplets. The aerosol generation mechanism of the Blaustein atomizer does not involve recirculation and concentration of the feed over time, which ensures a controlled and consistent output of bacterial aerosols (35). The Blaustein atomizer can operate at airflow rates ranging from 0.5 to 3.6 L/min and feed flow rates from 100 μL/min to 6 mL/min (35). To investigate the impact of airflow rate on aerosol properties, we generated droplets using airflow rates of 0.5, 1.5, and 2.5 L/min (Fig. 5). Table S15 provides the operating values held constant during these trials. Tables S16–S18 provide stage-specific viable bacteria-laden droplet count data using varied bacterial feed suspensions.

The mean size of viable bacteria-laden droplets showed a significant decrease with increasing flow rates over a range of 4.01 ± 0.16, 3.13 ± 0.10, and 2.74 ± 0.04 μm (Fig. 5a). Viable bacteria-laden droplet counts increased from 560 ± 143, 2,232 ± 346, to 3,309 ± 653 for 0.5, 1.5, and 2.5 L/min airflow rates, respectively (Fig. 5b). Figure 5c presents the percentage of viable bacteria-laden droplets collected below each stage under varied atomizer airflow rates. The highest percentages of viable bacteria-laden droplets generated using a 0.5 L/min atomizer airflow rate were collected below stages 2 and 3 with cutoff diameters of 4.7 and 3.3 μm, respectively (Fig. 5c). Meanwhile, the highest percentages of viable bacteria-laden droplets generated using a 1.5 L/min atomizer airflow rate were collected below stages 3 and 4 with cutoff diameters of 3.3 and 2.1 μm, respectively (Fig. 5c). Lastly, the highest percentages of viable bacteria-laden droplets generated using a 2.5 L/min atomizer airflow rate were collected below stages 4 and 5 cutoff diameters of 2.1 and 1.1 μm, respectively (Fig. 5c). Thus, as the atomizer airflow rate increases, viable bacteria-laden droplets are left-skewed and collect primarily on stages with lower cutoff values (Fig. 5c). Notably, an atomizer airflow rate of 1.5 L/min was the only condition that produced viable bacteria-laden droplets with mean size and count within the ASTM-specified range, as indicated by the shaded regions in Fig. 5a and b, respectively.

Increases in atomizer airflow rates were associated with a decrease in mean size (Fig. 5a) and a corresponding increase in the total count of viable bacteria-laden droplets (Fig. 5b). These trends align with prior reports in the literature, highlighting an inverse relationship between droplet size and airflow rates during jet atomization (36). The Weber number governs the process of liquid jet atomization by high-velocity gas streams (37). The nondimensional Weber number is the ratio between the deforming force created by the gas stream and the restoring surface tension force of the liquid stream (37). The aerodynamic Weber number $W_{e}$ for low-viscosity liquids can be calculated according to Eq. 10, where $\rho_{g}$ (Pa·s) is the gas density, $U_{g}$ (m/s) is the gas velocity, $D_{l}$ (m) is the diameter of the liquid jet, and *σ* (N/m) is the surface tension of the liquid (37).


(10) $$W_{e}=\frac{\rho_{g}U_{g}^{2}D_{l}}{\sigma }$$

Higher airflow velocities ($U_{g}$) produce significant deformations in the liquid stream, corresponding to a higher Weber number and finer droplet sizes (37). As discussed previously, smaller droplets are less likely to be removed by impaction and sedimentation, which explains the corresponding increase in droplet counts with decreased droplet size (Fig. 5).

### Effect of atomizer feed flow rate on viable bacteria-laden aerosol properties

**Fig. 6.**
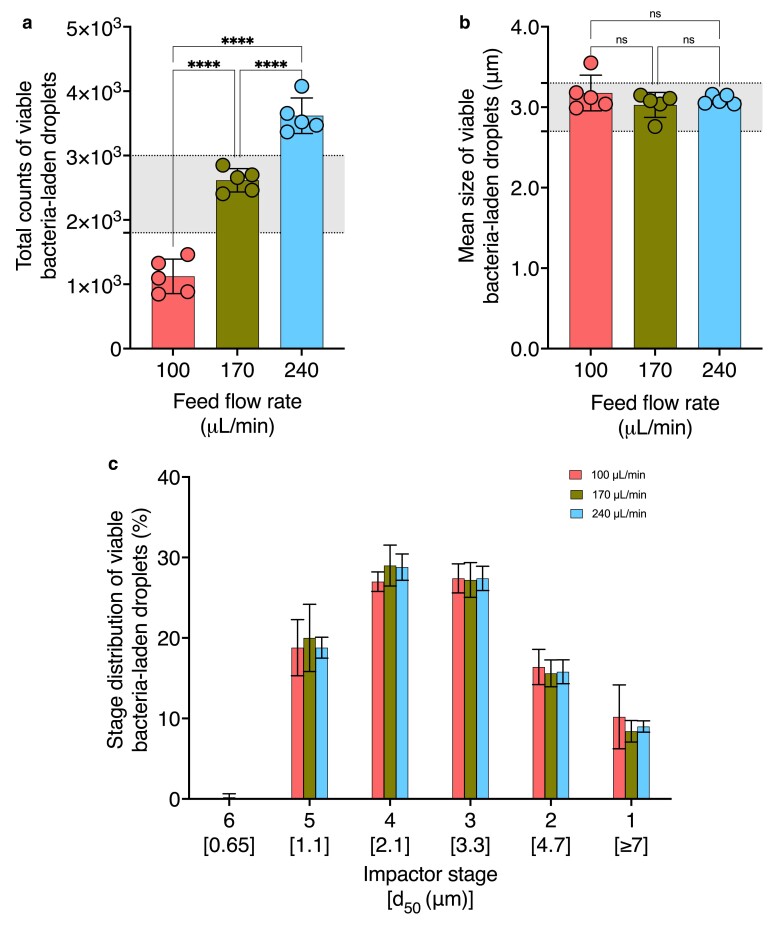
Effect of atomizer feed flow rates on mean size, total counts, and stage distribution of viable bacteria-laden droplets. The impact of varying atomizer feed flow rates on a) mean size, b) total count, and c) stage distribution of viable bacteria-laden droplets is shown (*n* = 5). All other operating variables for aerosol generation were kept constant according to Table S19 as the atomizer feed flow rate was varied. The remaining parameters were adjusted as specified in Table 1. Regions shaded in gray indicate the ASTM standard F2101-19-specified range for a) mean size and b) total count of viable bacteria-laden droplets. Asterisks indicate statistically significant differences, defined as *P-value < 0.05, **P-value <0.01, ***P-value <0.001, ****P-value <0.0001.

To investigate the impact of bacterial feed delivery rate on aerosol properties, we generated droplets using feed flow rates of under 100, 170, and 240 μL/min (Fig. 6). Table S19 provides the operating values held constant during these trials. Tables S20–S22 provide stage-specific viable bacteria-laden droplet count data under varied atomizer feed flow rates. The mean size of viable bacteria-laden droplets ranged from 3.18 ± 0.22, 3.03 ± 0.16, to 3.09 ± 0.06 μm for 100, 170, and 240 μL/min flow rate, respectively, showing no significant change (Fig. 6a). The total count of viable bacteria-laden droplets showed an increase in the range of 1,123 ± 269, 2,615 ± 181, and 3,619 ± 276 for 100, 170, and 240 μL/min flow rate, respectively (Fig. 6b). Figure 6c presents the stage distribution of viable bacteria-laden droplets. The generation of droplets under the three feed flow rates all produce similar stage distribution patterns, with the highest fraction of viable bacteria-laden droplets collected below stages 3 and 4 (Fig. 6c). Among the three conditions tested, an atomizer feed flow rate of 170 μL/min produced viable bacteria-laden aerosols within the ASTM-specified size and count range, as indicated by the shaded regions in Fig. 6a and b, respectively. Under an increased feed flow rate, atomization of a larger volume of the bacterial suspension generates more droplets (Fig. 6b). Our findings are consistent with data reported by Yi et al. (38), showing an increase in the viable droplet counts with an increase in the feed flow rate from 0.1 to 0.9 mL/min.

### BFE and breathability of test materials

**Fig. 7.**
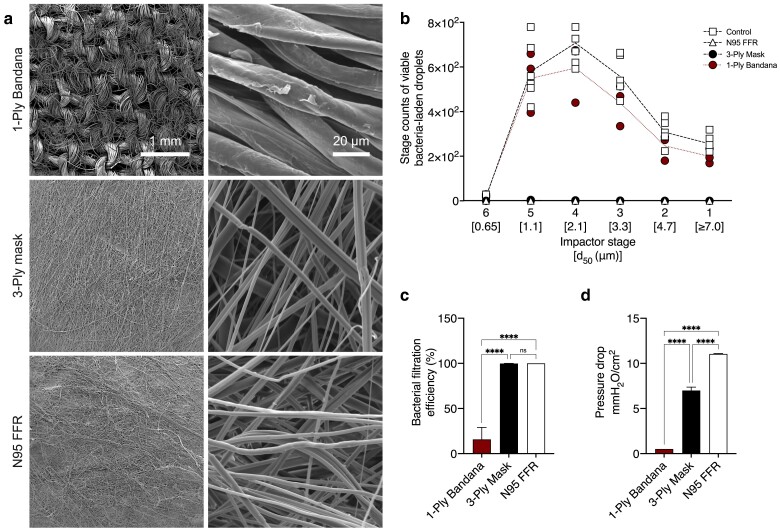
Dependence of BFE and breathability on test material. a) Scanning electron microscope images of 1-ply bandana, 3-ply mask (filter layer), and N95 face filtering respirator (filter layer). b) Bacteria-laden droplets collected below impactor stages in the presence and absence of test materials are shown (*n* = 3). Dotted lines are added to guide the eye and have no scientific significance. c) BFE, and d) pressure differential of test materials (*n* = 3). Significance was defined as *P < 0.05, **P < 0.01, ***P < 0.001, ****P < 0.0001. These operating values generate bacterial challenge consisting of 1,700 to 3,000 viable bacteria-laden droplets with a mean size of 2.7 to 3.3 μm, ≥80% relative humidity, 1.5% w/v peptone water concentration, 1 × 10^5^ CFU/mL bacterial concentration, and 1.5 L/min and 170 μL/min atomizer airflow and feed flow rates, respectively.

We applied our findings to generate a bacterial aerosol challenge with the ASTM-specified size and count range to perform BFE testing of selected materials (Tables S23–S30). Specifically, the following test values consistently produced between 1,700 and 3,000 viable bacteria-laden droplets with a mean aerodynamic size range of 2.7 to 3.3 µm across 25 aerosol trials conducted over 12 months: 1.5% w/v peptone water concentration, ≥ 80% relative humidity at 24 ± 2 °C, 1 × 10^5^ CFU/mL bacterial feed suspension concentration, 1.5 L/min atomizer airflow rate, and 170 µL/min feed flow rate (Table S30, Fig. S3). The microscopic fiber arrangements of three mask materials, visualized by SEM, are shown in Fig. 7a. Figure 7b shows the size and quantity of bacterial droplets detected in the presence and absence of mask materials. The BFE was calculated using the measured droplet counts for the 1-ply bandana, 3-ply mask, and N95 FFR, resulting in values of 15.76 ± 13.27, 99.86 ± 0.11, and 100.00%, respectively (Fig. 7c). Microbial growth was absent on all negative control plates conducted at the end of the experimental period. Additionally, the average pressure drop across the 1-ply bandana, 3-ply mask, and N95 FFR at an airflow velocity of 27.2 cm/s was also measured, resulting in resistance values of 0.52 ± 0.05, 7.00 ± 0.37, and 11.04 ± 0.05 mmH_2_O/cm^2^, respectively (Fig. 7d).

The effectiveness of face masks in safeguarding against airborne transmission hinges on the filtration quality of the material. The 1-ply cotton bandana exhibited poor filtration efficiency, with an average BFE value of 15.76 ± 13.27% (Fig. 7c). The BFE results for the 1-ply bandana align with the findings of others, including Konda et al (39), who reported a filtration efficiency of 14% for single-layer cotton material with a low thread count when challenged with sodium chloride particles 0.3 to 6 µm in size (34). The bandana demonstrated high breathability (Fig. 7d), which is expected based on the large mesh size observed in the electron micrograph images (Fig. 7a). In contrast, the filtration performance of 3-ply masks was excellent, with an average BFE of 99.86 ± 0.11% (Fig. 7c), albeit with a moderately high-pressure drop of 7.00 ± 0.37 mmH_2_O/cm^2^ (Fig. 7d). The ASTM barrier level 1 designation identifies masks with BFE ≥95% and pressure differential <5.1 mmH_2_O/cm^2^, while barrier level 2 and 3 designations are for masks with BFE ≥98% and pressure differential <6.1 mmH_2_O/cm^2^ (6). The 3-ply masks evaluated in this study fulfilled the filtration requirement but failed to meet the pressure differential for the ASTM barrier designations.

The N95 FFRs were used to benchmark superior mask filtration performance. The NIOSH-approved N95 FFRs, designed to filter out at least 95% of 0.3-µm-sized sodium chloride particles, represent a more stringent test than the BFE method (17). Our filtration test results for N95 FFRs align with expectations for BFE, consistent with previously reported values obtained using the ASTM platform (17, 40). The breathability of the N95 FFR material was poor as compared to the other samples; however, the measured pressure differential of 11.04 ± 0.05 mmH_2_O/cm^2^ is within the accepted maximum values of 35 and 25 mmH_2_O/cm^2^ during inhalation and exhalation, respectively.

## Implications

Reliable testing of mask filtration quality requires infectious droplet generation of realistic size and number that wearers might encounter in the environment. Aerodynamic size dictates whether infectious aerosol droplets reach the airways of a potential host (41, 42). Larger droplets, with increased settling velocities, are less likely to be inhaled to mediate airborne disease transmission (41, 43). For instance, 1, 5, and 10 μm droplets falling from a height of 2 m will reach the ground in 18.5 h, 45 min, and 11 min, respectively (41). Droplets in the 1 to 5 μm size range are more likely to be inhaled due to their longer airborne residence time. The ASTM-specified size range falls within these calculated bounds, underscoring the relevance of the bacterial aerosol challenge size range for simulating airborne transmission scenarios.

Regarding infectious droplet counts, studies have reported an elevation in exhaled droplet counts from SARS-CoV-2 patients (44, 45). Specifically, PCR-positive SARS-CoV-2 patients exhaled 1,490 droplets per liter (d/L), while PCR-negative individuals exhaled only 252 d/L (44). Despite the observed increase in total exhaled droplets during an active SARS-CoV-2 infection, what fraction of these droplets are infectious remains unclear. The average ventilation rate for humans at rest is approximately 6 L/min (46). An estimated 2,700 infectious droplets are exhaled over 2 min if we assume 10% are infectious. This theoretical estimate, based on clinical evidence, aligns with the ASTM-specified droplet count range used to establish filtration efficiencies of mask materials (8).

Our findings highlight the range of operating conditions for aerosol generation that successfully produce aerosols within the size and count range specified by the ASTM Guidance document. Inadequate control of the test conditions can lead to erroneous BFE measurements and inconsistent results between labs. Manipulating operating variables, including suspension media concentration, relative humidity, bacterial concentration, atomizer airflow, and feed flow rates, revealed trends in bacterial aerosol size and counts. We identified established theories to support the mechanisms proposed for the changes in droplet size and count with parameter changes, as summarized in Fig. S4. Certain variables, such as RH, are dictated by environmental conditions and geographical location and may be beyond the control of a standard test or a research laboratory unless they invest in an environmentally controlled room or biological safety cabinet. Consistent aerosol generation achieved using optimized test parameters led to BFE values consistent with existing literature, indicating our ability to ensure reproducibility under standard conditions.

## Supplementary Material

pgae473_Supplementary_Data

## Data Availability

All data generated in this study are included in the supporting information and Excel dataset files.
