# Supplementary material for: Unraveling the impact of operational parameters and environmental conditions on the quality of viable bacterial aerosols
Source: PNAS Nexus. 2024 Oct 30;3(11):pgae473. doi: 10.1093/pnasnexus/pgae473 (PMC11551483; doi:10.1093/pnasnexus/pgae473)
Supplement: pgae473_Supplementary_Data [file pgae473_supplementary_data.docx]

**Supporting information**

Unravelling the impact of operational parameters and environmental conditions on the quality of viable bacterial aerosols

*Mathura Thirugnanasampanthar^1^, Lei Tian^1^, Rod G Rhem^2^, Danielle D Libera^3^, Mellissa Gomez^1^, Kyle Jackson^1,4^, Alison E Fox-Robichaud^5,6^, Myrna B Dolovich^4,5,6*^ & Zeinab Hosseinidoust^1,4,6,7,8*^*

^1^McMaster University, Department of Chemical Engineering, 1280 Main Street West, Hamilton, ON, L8S 4L7, Canada

^2^Affiliate, Research Institute of St Joseph’s Hospital and Firestone Institute for Respiratory Health, 50 Charlton Ave East, Hamilton, ON, L8N 4A6, Canada

^3^McMaster University, Department of Biochemistry and Biomedical Sciences, 1280 Main Street West, Hamilton, ON, L8S 4K1, Canada

^4^McMaster University, Farncombe Family Digestive Health Research Institute, Hamilton, ON, L8S 4K1, Canada

^5^McMaster University, Faculty of Health Sciences, Department of Medicine, 1200 Main Street West, Hamilton, ON, L8N 3Z5, Canada

^6^McMaster University, Centre of Excellence in Protective Equipment and Materials, 1280 Main Street West, Hamilton, ON, L8S 4L7, Canada

^7^McMaster University, School of Biomedical Engineering, 1280 Main Street West, Hamilton, ON, L8S 4L7, Canada

^8^McMaster University, Michael DeGroote Institute for Infectious Disease Research, 1280 Main Street West, Hamilton, ON, L8S 4L8, Canada

*Corresponding Authors. ZH: [doust@mcmaster.ca](mailto:doust@mcmaster.ca); MD: mdolovic@mcmaster.ca

**Table of Contents**

Section S1. Mean aerodynamic size and total count determination of viable bacteria-laden aerosols

Section S2. Operating conditions

Section S3. Loss quantification within the system

Section S4. Peptone water concentration effects on viable bacteria-laden aerosol properties

Section S5. Relative humidity effects on viable bacteria-laden aerosol properties

Section S6. Pathogen concentration effects on viable bacteria-laden aerosol properties

Section S7. Atomizer air flow rate effects on viable bacteria-laden aerosol properties

Section S8. Atomizer feed flow rate effects on viable bacteria-laden aerosol properties

Section S9. Bacterial filtration efficiency trials

Section S10. Stage distribution of viable bacteria-laden droplets under optimized test conditions

Dataset 1. All data generated in this study is additionally provided in the Excel dataset file

Number of pages: 17

Number of tables: 30

Number of figures: 4

**SECTION S1. MEAN AERODYNAMIC SIZE AND TOTAL COUNT DETERMINATION OF VIABLE BACTERIA-LADEN AEROSOLS**

The six-stage viable cascade impactor was selected as the bioaerosol sampler for use within the test setup to determine the total counts and mean aerodynamic size of viable bacteria-laden droplets because the ASTM standard F2101-19 refers to this instrument **(Table 1)**.^1^ Moreover, among agar-based impaction systems for bioaerosol sampling, the Andersen six-stage viable cascade impactor has become a commonly used reference sampler since its introduction in 1958.^2^

**Mean aerodynamic size of viable bacteria-laden droplets**

The aerodynamic size reflects the behaviour of the droplet in a gaseous medium such as air and results from the size, shape, and density.^3^ Each impactor stage contains 400 perforations, forming 11 concentric rings. Agar plates are positioned beneath each stage to collect droplets through impaction. Aerosols that travel through perforations will either maintain a downward trajectory until they strike the plate or follow the air currents around the glass Petri dishes to the next stage. Aerosols with sufficient momentum cannot deviate from the downward trajectory and impact the collection medium. Conversely, smaller aerosols deviate from their initial downward trajectory and follow a curved trajectory around the plates towards lower stages.

Stage cut-off diameter indicates the size of droplets collected with 50% efficiency below a given stage. Collection efficiencies increase above 50% for larger droplets and decrease below 50% for smaller droplets relative to the stage cut-off diameter. Notably, the diameter of stage perforations decreases from the first to the sixth stage: 1.18, 0.91, 0.71, 0.53, 0.34, and 0.25 mm.^4^ Consequently, airflow velocity through lower stage perforations must increase proportionally for the volumetric flow rate to remain constant within the impactor. Smaller droplets that escape collection gain momentum with higher airflow velocities experienced on lower stages. Stage cut-off diameter decreases from the first to the sixth stage: 7.0, 4.7, 3.3, 2.1, 1.1, and 0.65 µm.^4^ Thus, smaller aerosols are collected beneath lower stages as they require higher velocities to achieve sufficient momentum for impaction. Conversely, larger aerosols with sufficient momentum collect below the upper stages.

**Total counts of viable bacteria-laden droplet**

Viable bacterial-laden droplet counts reflect the colonies-forming units (CFU) detected on tryptic soy agar (Figure S1). Viable bacteria-laden droplets that impact the agar medium produce colonies. Droplets travel through one of 400 stage perforations to impact the collection medium below. An underestimate of the number of viable bacteria-laden droplets can result from multiple viable bacteria-laden droplets entering through the same perforation to produce a single colony. Therefore, a correction was applied to the ‘raw’ colony counts, accounting for the masking effect of multiple viable bacteria-laden droplets impacting the same region.^5,6^ Reliable estimation of viable bacteria-laden droplet counts is only possible when there are less than 400 bacterial colonies per plate. Trials performed with 1×10^6^ CFU/mL suspensions produced 400 colonies on five of the six collection plates; therefore, the viable bacteria-laden droplet counts presented could be an underestimation of the actual value.

**Aerosol trials performed in this work**

5 parameters x 3 test value per parameter = 15 conditions

15 conditions x 5 replicates per condition = 75 runs performed, analysed, and graphed (excluding 3 replicate BFE trials)

**Aerosol trials required for all permutations**

5 parameters with 3 test value = 3^5^ total permutations = 243 conditions

243 conditions x 3 replicates = 729 runs required

243 conditions x 5 replicates = 1215 runs required

**Figure S1**. Scan of single aerosol run collection plate.

**SECTION S2. OPERATING CONDITIONS**

**Preparation and delivery of bacterial feed suspension**

Bacterial culture preparation used 10 µL of 25% v/v *S. aureus* glycerol stock stored at -80°C, introduced with an inoculation loop (VWR 89126-870), into 3 mL of fresh TSB media in a snap cap culture tube (Fisherbrand 14-956-9B). Bacteria were grown for 18 h at 37°C in a shaking incubator set to perform 180 rotations per minute. Bacterial cultures were stored at 4ºC before use and were discarded one week after preparation. An appropriate volume of the bacterial culture, with a concentration of 1×10^9^ CFU/mL, was added to 10 mL aliquot of peptone water solution to prepare the feed suspension (1×10^4^, 1×10^5^, or 1×10^6^ CFU/mL).

Bacterial suspension in peptone water was dispensed from a 5 mL syringe (BD 309646) and delivered to the atomizer using a 1/16’’ x 3/16’’ ID/OD silicone tubing (McMaster-Carr 51135K608). Feed was delivered to the atomizer at a controlled feed flow rate (100, 170, or 240 µL/min) for 1 min using a syringe pump (Fisherbrand 780100I). Bacterial feed concentration was verified using the dilution plating method. Briefly, the feed suspension was serially diluted in 900 µL aliquots of 0.9% (w/v) saline, followed by the plating of 100 µL aliquots from an appropriate dilution on TSA plates in triplicates. Plates were incubated at 37°C for 24 h to allow for colony formation. Average colony counts from the three plates were used to verify the concentration of the bacterial feed used to generate viable bacteria-laden aerosol droplets.

**Test values selected for unspecified operating conditions**

For the unspecified operating conditions highlighted in **Table 1**, we investigated the effect of three test values on the quality of the generated bacterial aerosol. The ambient temperature within the biosafety cabinet (BSC), housing the aerosol exposure platform, was monitored and remained relatively stable over a year-long period ranging between 22.4 and 25.6°C. We could not freely manipulate the temperature within the BSC, which excluded this variable from our investigation. We examined three relative humidity ranges: low (≤20%), intermediary (40-60%), and high (≥80%), at ambient temperature conditions between 22.4 to 25.6°C. The bacterial suspension concentration, starting at 1×10^4^ CFU/mL, was increased by 1-log increments up to 1×10^6^ CFU/mL. The lowest atomizer airflow and feed flow values investigated reflect the minimum operating requirements for the Blaustein single-jet atomizer module. The airflow rate was increased by 1 L/min increments in 3 steps starting from 0.5 L/min. Meanwhile, the feed flow rate, starting at 100 µL/min, was increased by 70 µL/min in 3-step increments.

The relative humidity of the makeup air controls the relative humidity within the aerosol platform. The makeup air (27.8, 26.8, 25.8 L/min) is filtered room air that is passed through an inline HEPA capsule filter (TSI 1602051) before being drawn into the 60-cm-high glass aerosol mixing chamber and mixes with aerosol droplets before being drawn into the impactor by the downstream vacuum pump (**Figure 1**). We performed the ≤20% RH runs during winter when ambient RH conditions were low, producing ≤20% RH airflows within the platform. We performed the 40-60% RH runs during the summer with intermediate RH conditions, producing airflow with a 40-60% RH within the platform. For the ≥80 RH runs, we passed filtered room air through an inline water-to-air humidifier before it entered the 60-cm-high aerosol mixing chamber, and airflow within the platform reached ≥80% RH. We confirmed the RH of the airflow within the platform by placing the SensorPush device between the stages of an assembled impactor without the agar plates (just assembled stages) and conducting an airflow-only run (without atomizing the bacterial feed) before each trial.

**Differential pressure testing conditions**

The differential pressure (DP) test assesses resistance to airflow across the test material. Potassium sulphate was dissolved in 500 mL of deionized water to prepare a saturated salt solution and placed inside a sealed bag with the test materials. A PushSensor thermometer-hygrometer (HT.w 16794383) placed inside the sealed bag was used to ensure masks were conditioned for a minimum of 4 h at 85±5% RH at 21±5ºC before DP or BFE tests. DP tests measure the change in pressure across the material under constant airflow. The differential pressure tests, performed according to standard specifications, subjects the material to 27 cm/s airflow velocity, while the BFE testing subjects the material to 9.6 cm/s airflow velocity.^1,7,8^ A device was constructed according to specification in British Standard BS EN 14683:2019 + AC:2019 to measure DP across test materials.^7,8^ The conditioned test material was placed within the device holder with an area of 4.9 cm^2^ and oriented to simulate exhalation. DP was measured using a differential manometer (Dwyer Instruments 2310) at an airflow rate of 8 L/min across the material. An average of five DP values from different regions of each test material provides a representative value.

**SECTION S3. LOSS QUANTIFICATION WITHIN THE SYSTEM**

1. Values of operating conditions for loss quantification trial

| Peptone water concentration  (% w/v) | Relative humidity (%) | Fluorescein concentration (mg/mL) | Atomizer airflow rate (L/min) | Atomizer feed flow rate (µL/min) |
| --- | --- | --- | --- | --- |
| 1.5, 3, 6 | ≤20 | 1 | 1.5 | 170 |

A droplet loss quantification trial was performed using 1 mg/mL sodium fluorescein (Sigma-Aldrich F6377-100G) added to 1.5, 3, and 6% w/v peptone water solutions free of bacteria and atomized under ≤20% RH humidity conditions. A 170 µL volume of the fluorescein-containing peptone water solution was atomized over one minute using 1.5 LPM of compressed airflow. Foils were placed over 27 mL tryptic soy agar-containing glass Petri dishes to collect the droplets. The deposits were removed by washing the foils in 5 mL ultrapure water. Spectrophotometric readings of fluoresceine samples were taken at excitation and emission wavelengths of 490±5 and 515±5 nm, respectively (Biotek Synergy Neo2) of 200 µL of wash samples in 96-well microplates (Greiner Bio-One 655086).

The difficulty in removing the fluorescein dye from the inner surfaces of the system (impactor stages, 60-cm-high glass aerosol mixing chamber, atomizer nozzle) discouraged additional trials. The data demonstrate that 18%, 13%, and 6.5% of the fluorescein-containing feed, introduced into the system via atomization, was recovered from below the impactor stages. The loss within the system for the 6% w/v peptone water solution is more than double that of the 1.5% w/v peptone water solution.

1. Nominal doses (%) of fluoresceine-containing peptone water solutions

| Stage | Cut-off (µm) | Recovered dose  1.5% w/v | Recovered dose  3% w/v | Recovered dose  6% w/v |
| --- | --- | --- | --- | --- |
| 1 | ≥7 | 2.17 | 2.27 | 1.21 |
| 2 | 4.7 | 2.01 | 1.92 | 0.94 |
| 3 | 3.3 | 1.97 | 2.03 | 2.01 |
| 4 | 2.1 | 0.82 | 2.98 | 0.64 |
| 5 | 1.1 | 5.33 | 2.67 | 1.25 |
| 6 | 0.65 | 5.96 | 1.15 | 0.44 |
| Recovered (%) | | 18.26 | 13.01 | 6.49 |
| Loss (%) | | 81.74 | 86.99 | 93.51 |

**Figure S2**. Stage distribution of the nominal doses of fluorescein-containing droplets.

**SECTION S4. PEPTONE WATER CONCENTRATION EFFECTS ON VIABLE BACTERIA-LADEN AEROSOL PROPERTIES**

1. Figure 2: Values for operating parameters under varied peptone water concentration.

| Peptone water concentration  (% w/v) | Relative humidity (%) | Bacterial concentration (CFU/mL) | Atomizer airflow rate (L/min) | Atomizer feed flow rate (µL/min) |
| --- | --- | --- | --- | --- |
| 1.5, 3, 6 | ≥80 | 1×10^5^ | 1.5 | 170 |

1. Figure 2 Data Set A: Droplet counts for five independent aerosol trials performed with 1.5% w/v peptone water suspension media.

| d_50_ | N_R_ | **N_CC_** | N_R_ | N_CC_ | N_R_ | N_CC_ | N_R_ | N_CC_ | N_R_ | N_CC_ |
| --- | --- | --- | --- | --- | --- | --- | --- | --- | --- | --- |
| 7 | 176 | **232** | 170 | 221 | 135 | 165 | 129 | 156 | 132 | 160 |
| 4.7 | 268 | **444** | 215 | 308 | 233 | 349 | 251 | 395 | 248 | 387 |
| 3.3 | 321 | **649** | 312 | 606 | 260 | 420 | 318 | 634 | 307 | 584 |
| 2.1 | 343 | **779** | 331 | 703 | 328 | 686 | 326 | 675 | 314 | 615 |
| 1.1 | 341 | **766** | 344 | 786 | 274 | 462 | 288 | 509 | 292 | 524 |
| 0.65 | 25 | **26** | 24 | 25 | 4 | 4 | 4 | 4 | 2 | 2 |
| MPS |  | **2.88** |  | 2.78 |  | 2.94 |  | 2.96 |  | 2.96 |
| Total |  | **2896** |  | 2649 |  | 2086 |  | 2373 |  | 2272 |

d_50_ = Stage cut-off diameter; N_R_ = Raw counts; N_C_ = Coincidence-correct counts; MPS = Mean particle size (µm)

**S2 - Sample calculations**

$${Mean droplet size, \bar{D}}_{\mathrm{ae}}=\frac{\sum_{i=1}^{6} d_{50,i}\times N_{c,i}}{N_{T}}$$

$$= \frac{\left[ 7\times232+4.7\times444+3.3\times649+2.1\times779+1.1\times766+0.65\times26 \right]}{\left[ 232+444+649+779+766+26 \right]}$$

$$=2.88 \mu m$$

$$N_{T}=\sum_{i=1}^{6} N_{c,i}$$

$$Total droplet counts, N_{T}= 232+444+649+779+766+26$$

$$=2896$$

1. Figure 2 Data Set B: Droplet counts for five independent aerosol trials performed with 3% w/v peptone water suspension media.

| d_50_ | N_R_ | N_C_ | N_R_ | N_C_ | N_R_ | N_C_ | N_R_ | N_C_ | N_R_ | N_C_ |
| --- | --- | --- | --- | --- | --- | --- | --- | --- | --- | --- |
| 7 | 188 | 254 | 205 | 287 | 186 | 250 | 188 | 254 | 186 | 250 |
| 4.7 | 292 | 524 | 298 | 547 | 274 | 462 | 287 | 506 | 268 | 444 |
| 3.3 | 340 | 759 | 367 | 998 | 336 | 733 | 343 | 779 | 340 | 759 |
| 2.1 | 353 | 857 | 354 | 865 | 326 | 675 | 326 | 675 | 327 | 680 |
| 1.1 | 248 | 387 | 238 | 362 | 229 | 340 | 240 | 367 | 205 | 287 |
| 0.65 | 1 | 1 | 1 | 1 | 3 | 3 | 2 | 2 | 1 | 1 |
| MPS |  | 3.22 |  | 3.30 |  | 3.30 |  | 3.31 |  | 3.34 |
| Total |  | 2782 |  | 3060 |  | 2463 |  | 2583 |  | 2421 |

1. Figure 2 Data Set C: Droplet counts for five independent aerosol trials performed with 6% w/v peptone water suspension media.

| d_50_ | N_R_ | N_C_ | N_R_ | N_C_ | N_R_ | N_C_ | N_R_ | N_C_ | N_R_ | N_C_ |
| --- | --- | --- | --- | --- | --- | --- | --- | --- | --- | --- |
| 7 | 178 | 236 | 182 | 243 | 192 | 262 | 176 | 232 | 176 | 232 |
| 4.7 | 279 | 478 | 300 | 555 | 301 | 559 | 280 | 482 | 284 | 495 |
| 3.3 | 326 | 675 | 327 | 680 | 327 | 680 | 328 | 686 | 330 | 697 |
| 2.1 | 282 | 488 | 288 | 509 | 308 | 588 | 261 | 423 | 287 | 506 |
| 1.1 | 89 | 101 | 149 | 186 | 182 | 243 | 60 | 65 | 100 | 115 |
| 0.65 | 0 | 0 | 0 | 0 | 0 | 0 | 0 | 0 | 0 | 0 |
| MPS |  | 3.67 |  | 3.60 |  | 3.52 |  | 3.77 |  | 3.64 |
| Total |  | 1978 |  | 2173 |  | 2332 |  | 1888 |  | 2045 |

**SECTION S5. RELATIVE HUMIDITY EFFECTS ON VIABLE BACTERIA-LADEN AEROSOL PROPERTIES**

1. Figure 3: Values for operating parameters under varied relative humidity.

| Peptone water concentration  (% w/v) | Relative humidity (%) | Bacterial concentration (CFU/mL) | Atomizer airflow rate (L/min) | Atomizer feed flow rate (µL/min) |
| --- | --- | --- | --- | --- |
| 1.5 | ≤20,40-60, ≥80 | 1×10^5^ | 1.5 | 170 |

1. Figure 3 Data Set A: Droplet counts for five independent aerosol trials performed at ≤20% relative humidity.

| d_50_ | N_R_ | N_C_ | N_R_ | N_C_ | N_R_ | N_C_ | N_R_ | N_C_ | N_R_ | N_C_ |
| --- | --- | --- | --- | --- | --- | --- | --- | --- | --- | --- |
| 7 | 216 | 311 | 207 | 292 | 206 | 289 | 232 | 347 | 239 | 364 |
| 4.7 | 262 | 426 | 308 | 588 | 315 | 620 | 300 | 555 | 334 | 721 |
| 3.3 | 300 | 555 | 362 | 942 | 358 | 902 | 350 | 832 | 375 | 1109 |
| 2.1 | 380 | 1198 | 383 | 1263 | 387 | 1371 | 391 | 1518 | 397 | 1961 |
| 1.1 | 374 | 1093 | 383 | 1263 | 383 | 1263 | 393 | 1619 | 394 | 1681 |
| 0.65 | 55 | 59 | 49 | 52 | 65 | 71 | 82 | 92 | 88 | 99 |
| MPS |  | 2.68 |  | 2.73 |  | 2.71 |  | 2.58 |  | 2.63 |
| Total |  | 3642 |  | 4400 |  | 4516 |  | 4963 |  | 5935 |

1. Figure 3 Data Set B: Droplet counts for five independent aerosol trials performed at 40-60% relative humidity.

| d_50_ | N_R_ | N_C_ | N_R_ | N_C_ | N_R_ | N_C_ | N_R_ | N_C_ | N_R_ | N_C_ |
| --- | --- | --- | --- | --- | --- | --- | --- | --- | --- | --- |
| 7 | 202 | 281 | 196 | 269 | 166 | 214 | 185 | 248 | 176 | 232 |
| 4.7 | 326 | 675 | 317 | 629 | 268 | 444 | 291 | 520 | 288 | 509 |
| 3.3 | 365 | 975 | 368 | 975 | 326 | 675 | 343 | 779 | 329 | 692 |
| 2.1 | 376 | 1125 | 372 | 1064 | 347 | 809 | 342 | 772 | 344 | 786 |
| 1.1 | 359 | 911 | 374 | 1093 | 331 | 703 | 353 | 857 | 341 | 766 |
| 0.65 | 12 | 12 | 12 | 12 | 15 | 15 | 20 | 21 | 18 | 18 |
| MPS |  | 2.95 |  | 2.85 |  | 2.90 |  | 2.92 |  | 2.93 |
| Total |  | 3979 |  | 4042 |  | 2860 |  | 3197 |  | 3003 |

1. Figure 3 Data Set C: Droplet counts for five independent aerosol trials performed at ≥80% relative humidity.

| d_50_ | N_R_ | N_C_ | N_R_ | N_C_ | N_R_ | N_C_ | N_R_ | N_C_ | N_R_ | N_C_ |
| --- | --- | --- | --- | --- | --- | --- | --- | --- | --- | --- |
| 7 | 120 | 143 | 127 | 153 | 146 | 182 | 157 | 199 | 177 | 234 |
| 4.7 | 264 | 432 | 229 | 340 | 257 | 411 | 259 | 417 | 268 | 444 |
| 3.3 | 307 | 584 | 319 | 639 | 326 | 675 | 346 | 801 | 338 | 746 |
| 2.1 | 323 | 659 | 323 | 659 | 342 | 772 | 358 | 902 | 361 | 931 |
| 1.1 | 291 | 520 | 304 | 571 | 316 | 624 | 333 | 715 | 287 | 506 |
| 0.65 | 4 | 4 | 6 | 6 | 7 | 7 | 11 | 11 | 4 | 4 |
| MPS |  | 2.95 |  | 2.87 |  | 2.90 |  | 2.85 |  | 3.04 |
| Total |  | 2342 |  | 2368 |  | 2671 |  | 3045 |  | 2865 |

**SECTION S6. PATHOGEN CONCENTRATION EFFECTS ON VIABLE BACTERIA-LADEN AEROSOL PROPERTIES**

1. Figure 4: Values for operating parameters under varied bacterial concentration.

| Peptone water concentration  (% w/v) | Relative humidity (%) | Bacterial concentration (CFU/mL) | Atomizer airflow rate (L/min) | Atomizer feed flow rate (µL/min) |
| --- | --- | --- | --- | --- |
| 1.5 | ≥80 | 1×10^4^, 1×10^5^, 1×10^6^ | 1.5 | 170 |

1. Figure 4 Data Set A: Droplet counts for five independent aerosol trials performed with 1×10^4^ CFU/mL with bacterial concentration.

| d_50_ | N_R_ | N_C_ | N_R_ | N_C_ | N_R_ | N_C_ | N_R_ | N_C_ | N_R_ | N_C_ |
| --- | --- | --- | --- | --- | --- | --- | --- | --- | --- | --- |
| 7 | 20 | 21 | 11 | 11 | 12 | 12 | 12 | 12 | 10 | 10 |
| 4.7 | 68 | 75 | 20 | 21 | 27 | 28 | 18 | 18 | 24 | 25 |
| 3.3 | 114 | 134 | 44 | 47 | 43 | 46 | 55 | 59 | 46 | 49 |
| 2.1 | 93 | 106 | 51 | 55 | 64 | 70 | 93 | 106 | 81 | 91 |
| 1.1 | 100 | 115 | 55 | 59 | 59 | 64 | 66 | 72 | 67 | 73 |
| 0.65 | 2 | 2 | 0 | 0 | 1 | 1 | 0 | 0 | 1 | 1 |
| MPS |  | 2.85 |  | 2.65 |  | 2.65 |  | 2.49 |  | 2.49 |
| Total |  | 453 |  | 193 |  | 221 |  | 267 |  | 249 |

1. Figure 4 Data Set B: Droplet counts for five independent aerosol trials performed with 1×10^5^ CFU/mL with bacterial concentration.

| d_50_ | N_R_ | N_C_ | N_R_ | N_C_ | N_R_ | N_C_ | N_R_ | N_C_ | N_R_ | N_C_ |
| --- | --- | --- | --- | --- | --- | --- | --- | --- | --- | --- |
| 7 | 168 | 218 | 103 | 119 | 104 | 120 | 124 | 148 | 123 | 147 |
| 4.7 | 294 | 531 | 270 | 450 | 251 | 395 | 214 | 306 | 214 | 306 |
| 3.3 | 355 | 874 | 301 | 559 | 312 | 606 | 288 | 509 | 280 | 482 |
| 2.1 | 333 | 715 | 286 | 502 | 284 | 495 | 311 | 601 | 294 | 531 |
| 1.1 | 310 | 597 | 250 | 392 | 283 | 492 | 277 | 472 | 242 | 372 |
| 0.65 | 18 | 18 | 12 | 12 | 9 | 9 | 6 | 6 | 3 | 3 |
| MPS |  | 3.07 |  | 3.09 |  | 2.97 |  | 2.91 |  | 3.03 |
| Total |  | 2953 |  | 2034 |  | 2117 |  | 2042 |  | 1841 |

1. Figure 4 Data Set C: Droplet counts for five independent aerosol trials performed with 1×10^6^ CFU/mL with bacterial concentration.

| d_50_ | N_R_ | N_C_ | N_R_ | N_C_ | N_R_ | N_C_ | N_R_ | N_C_ | N_R_ | N_C_ |
| --- | --- | --- | --- | --- | --- | --- | --- | --- | --- | --- |
| 7 | 400 | 2628 | 400 | 2628 | 400 | 2628 | 400 | 2628 | 400 | 2628 |
| 4.7 | 400 | 2628 | 400 | 2628 | 400 | 2628 | 400 | 2628 | 400 | 2628 |
| 3.3 | 400 | 2628 | 400 | 2628 | 400 | 2628 | 400 | 2628 | 400 | 2628 |
| 2.1 | 400 | 2628 | 400 | 2628 | 400 | 2628 | 400 | 2628 | 400 | 2628 |
| 1.1 | 400 | 2628 | 400 | 2628 | 400 | 2628 | 400 | 2628 | 400 | 2628 |
| 0.65 | 16 | 16 | 143 | 177 | 122 | 146 | 231 | 345 | 217 | 313 |
| MPS |  | 3.64 |  | 3.60 |  | 3.61 |  | 3.56 |  | 3.57 |
| Total |  | 13156 |  | 13317 |  | 13286 |  | 13485 |  | 13453 |

**SECTION S7. ATOMIZER AIR FLOW RATE EFFECTS ON VIABLE BACTERIA-LADEN AEROSOL PROPERTIES**

1. Figure 5: Values for operating parameters under varied atomizer airflow rate.

| Peptone water concentration  (% w/v) | Relative humidity (%) | Bacterial concentration (CFU/mL) | Atomizer airflow rate (L/min) | Atomizer feed flow rate (µL/min) |
| --- | --- | --- | --- | --- |
| 1.5 | ≥80 | 1×10^5^ | 0.5, 1.5, 2.5 | 170 |

1. Figure 5 Data Set A: Droplet counts for five independent aerosol trials performed at 0.5 L/min atomizer airflow rate.

| d_50_ | N_R_ | N_C_ | N_R_ | N_C_ | N_R_ | N_C_ | N_R_ | N_C_ | N_R_ | N_C_ |
| --- | --- | --- | --- | --- | --- | --- | --- | --- | --- | --- |
| 7 | 113 | 133 | 86 | 97 | 73 | 81 | 98 | 112 | 99 | 114 |
| 4.7 | 160 | 204 | 107 | 125 | 88 | 99 | 135 | 165 | 121 | 144 |
| 3.3 | 144 | 179 | 112 | 131 | 89 | 101 | 165 | 213 | 135 | 165 |
| 2.1 | 104 | 120 | 68 | 75 | 64 | 70 | 109 | 127 | 110 | 129 |
| 1.1 | 48 | 51 | 26 | 27 | 19 | 19 | 67 | 73 | 44 | 47 |
| 0.65 | 0 | 0 | 0 | 0 | 0 | 0 | 1 | 1 | 0 | 0 |
| MPS |  | 4.06 |  | 4.15 |  | 4.14 |  | 3.78 |  | 3.91 |
| Total |  | 687 |  | 455 |  | 370 |  | 691 |  | 599 |

1. Figure 5 Data Set B: Droplet counts for five independent aerosol trials performed at 1.5 L/min atomizer airflow rate.

| d_50_ | N_R_ | N_C_ | N_R_ | N_C_ | N_R_ | N_C_ | N_R_ | N_C_ | N_R_ | N_C_ |
| --- | --- | --- | --- | --- | --- | --- | --- | --- | --- | --- |
| 7 | 139 | 171 | 144 | 179 | 138 | 169 | 136 | 166 | 209 | 296 |
| 4.7 | 252 | 398 | 202 | 281 | 277 | 472 | 209 | 296 | 272 | 456 |
| 3.3 | 328 | 686 | 319 | 639 | 313 | 610 | 301 | 559 | 320 | 644 |
| 2.1 | 297 | 543 | 286 | 502 | 340 | 759 | 300 | 555 | 343 | 779 |
| 1.1 | 203 | 283 | 224 | 328 | 279 | 478 | 245 | 379 | 293 | 527 |
| 0.65 | 0 | 0 | 1 | 1 | 4 | 4 | 0 | 0 | 0 | 0 |
| MPS |  | 3.26 |  | 3.16 |  | 3.02 |  | 3.06 |  | 3.17 |
| Total |  | 2081 |  | 1930 |  | 2492 |  | 1955 |  | 2702 |

1. Figure 5 Data Set C: Droplet counts for five independent aerosol trials performed at 2.5 L/min atomizer airflow rate.

| d_50_ | N_R_ | N_C_ | N_R_ | N_C_ | N_R_ | N_C_ | N_R_ | N_C_ | N_R_ | N_C_ |
| --- | --- | --- | --- | --- | --- | --- | --- | --- | --- | --- |
| 7 | 183 | 245 | 154 | 194 | 158 | 201 | 196 | 269 | 194 | 265 |
| 4.7 | 280 | 482 | 221 | 322 | 199 | 275 | 258 | 414 | 273 | 459 |
| 3.3 | 365 | 975 | 322 | 654 | 310 | 597 | 347 | 809 | 342 | 772 |
| 2.1 | 378 | 1160 | 332 | 709 | 333 | 715 | 370 | 1036 | 376 | 1125 |
| 1.1 | 370 | 1036 | 349 | 824 | 330 | 697 | 373 | 1078 | 378 | 1160 |
| 0.65 | 8 | 8 | 18 | 18 | 9 | 9 | 25 | 26 | 11 | 11 |
| MPS |  | 2.76 |  | 2.73 |  | 2.78 |  | 2.72 |  | 2.69 |
| Total |  | 3906 |  | 2721 |  | 2494 |  | 3632 |  | 3792 |

**SECTION S8. ATOMIZER FEED FLOW RATE EFFECTS ON VIABLE BACTERIA-LADEN AEROSOL PROPERTIES**

1. Figure 6: Values for operating parameters under varied atomizer feed flow rate.

| Peptone water concentration  (% w/v) | Relative humidity (%) | Bacterial concentration (CFU/mL) | Atomizer airflow rate (L/min) | Atomizer feed flow rate (µL/min) |
| --- | --- | --- | --- | --- |
| 1.5 | ≥80 | 1×10^5^ | 1.5 | 100, 170, 240 |

1. Figure 6 Data Set A: Droplet counts for five independent aerosol trials performed at 100 µL/min atomizer feed flow rate.

| d_50_ | N_R_ | N_C_ | N_R_ | N_C_ | N_R_ | N_C_ | N_R_ | N_C_ | N_R_ | N_C_ |
| --- | --- | --- | --- | --- | --- | --- | --- | --- | --- | --- |
| 7 | 116 | 137 | 188 | 254 | 73 | 81 | 61 | 66 | 74 | 82 |
| 4.7 | 158 | 201 | 172 | 225 | 111 | 130 | 120 | 143 | 168 | 218 |
| 3.3 | 250 | 392 | 258 | 414 | 178 | 236 | 167 | 216 | 207 | 292 |
| 2.1 | 240 | 367 | 240 | 367 | 185 | 248 | 174 | 228 | 209 | 296 |
| 1.1 | 175 | 230 | 158 | 201 | 151 | 190 | 153 | 193 | 161 | 206 |
| 0.65 | 0 | 0 | 0 | 0 | 0 | 0 | 2 | 2 | 0 | 0 |
| MPS |  | 2.85 |  | 2.65 |  | 2.65 |  | 2.49 |  | 2.49 |
| Total |  | 453 |  | 193 |  | 221 |  | 267 |  | 249 |

1. Figure 6 Data Set B: Droplet counts for five independent aerosol trials performed at 170 µL/min atomizer feed flow rate.

| d_50_ | N_R_ | N_C_ | N_R_ | N_C_ | N_R_ | N_C_ | N_R_ | N_C_ | N_R_ | N_C_ |
| --- | --- | --- | --- | --- | --- | --- | --- | --- | --- | --- |
| 7 | 140 | 172 | 168 | 218 | 166 | 214 | 175 | 230 | 190 | 258 |
| 4.7 | 231 | 345 | 234 | 352 | 256 | 409 | 267 | 440 | 273 | 459 |
| 3.3 | 330 | 697 | 313 | 610 | 336 | 733 | 343 | 779 | 335 | 727 |
| 2.1 | 334 | 721 | 342 | 772 | 332 | 709 | 328 | 686 | 356 | 883 |
| 1.1 | 335 | 727 | 270 | 450 | 251 | 395 | 291 | 520 | 291 | 520 |
| 0.65 | 36 | 38 | 4 | 4 | 2 | 2 | 3 | 3 | 3 | 3 |
| MPS |  | 2.76 |  | 3.04 |  | 3.15 |  | 3.11 |  | 3.08 |
| Total |  | 2700 |  | 2406 |  | 2462 |  | 2658 |  | 2850 |

1. Figure 6 Data Set C: Droplet counts for five independent aerosol trials performed at 240 µL/min atomizer feed flow rate.

| d_50_ | N_R_ | N_C_ | N_R_ | N_C_ | N_R_ | N_C_ | N_R_ | N_C_ | N_R_ | N_C_ |
| --- | --- | --- | --- | --- | --- | --- | --- | --- | --- | --- |
| 7 | 247 | 384 | 222 | 324 | 212 | 302 | 215 | 308 | 234 | 352 |
| 4.7 | 333 | 715 | 299 | 551 | 288 | 509 | 286 | 502 | 300 | 555 |
| 3.3 | 371 | 1050 | 357 | 892 | 358 | 902 | 372 | 1064 | 368 | 1010 |
| 2.1 | 382 | 1241 | 367 | 998 | 365 | 975 | 375 | 1109 | 361 | 931 |
| 1.1 | 328 | 686 | 330 | 697 | 326 | 675 | 325 | 670 | 325 | 670 |
| 0.65 | 1 | 1 | 10 | 10 | 5 | 5 | 2 | 2 | 4 | 4 |
| MPS |  | 3.16 |  | 3.07 |  | 3.05 |  | 3.04 |  | 3.15 |
| Total |  | 4077 |  | 3472 |  | 3368 |  | 3655 |  | 3522 |

**SECTION S9. BACTERIAL FILTRATION EFFICIENCY EVALUATIONS OF THREE TEST MATERIALS**

1. Figure 7: Values of optimized operating conditions.

| Peptone water concentration  (% w/v) | Relative humidity (%) | Bacterial concentration (CFU/mL) | Atomizer airflow rate (L/min) | Atomizer feed flow rate (µL/min) |
| --- | --- | --- | --- | --- |
| 1.5 | ≥80 | 1×10^5^ | 1.5 | 170 |

1. Figure 7b,7c Data Set A: Control aerosol trials.

| d_50_ | N_R_ Positive control A | **N_C_ Positive control A** | N_R_ Positive control B | **N_C_ Positive control B** | N_R_ Negative control A | N_C_  Negative control A |
| --- | --- | --- | --- | --- | --- | --- |
| 7 | 201 | **279** | 175 | **230** | 0 | 0 |
| 4.7 | 171 | **223** | 211 | **300** | 0 | 0 |
| 3.3 | 322 | **654** | 293 | **527** | 0 | 0 |
| 2.1 | 343 | **779** | 315 | **620** | 0 | 0 |
| 1.1 | 292 | **524** | 301 | **559** | 0 | 0 |
| 0.65 | 14 | **14** | 13 | **13** | 0 | 0 |
| MPS |  | **2.98** |  | **2.97** |  | - |
| Total |  | **2473** |  | **2249** |  | 0 |
| MPS | 2.97 | | | |  |  |
| Total | **2361** | | | |  |  |

**S24 - Sample calculation**

$${Total droplet counts,N}_{T}=\sum_{i=1}^{6} N_{c,i}$$

$$Total droplet counts, N_{T, A}= 279+223+527+620+559+13$$

$$=2473$$

$$Total droplet counts, N_{T,B}= 230+300+654+779+524+14$$

$$=2249$$

$$Average positive control droplet counts, N_{\mathrm{Avg}}= \frac{2473+2249}{2}$$

$$=2361$$

1. Figure 7b,7c Data Set A: Bacterial filtration efficiency trials.

| d_50_ | N_R_ Bandana | **N_C_ Bandana** | N_R_ 3-Ply Mask | N_C_ 3-Ply Mask | N_R_ N95 Respirator | N_C_ N95 Respirator |
| --- | --- | --- | --- | --- | --- | --- |
| 7 | 177 | **234** | 0 | 0 | 0 | 0 |
| 4.7 | 198 | **273** | 0 | 0 | 0 | 0 |
| 3.3 | 289 | **513** | 0 | 0 | 0 | 0 |
| 2.1 | 326 | **675** | 0 | 0 | 0 | 0 |
| 1.1 | 309 | **592** | 1 | 1 | 0 | 0 |
| 0.65 | 21 | **22** | 0 | 0 | 0 | 0 |
| MPS |  | **2.9** |  | 1.1 | - | - |
| Total |  | **2309** |  | 1 | 0 | 0 |
| BFE (%) |  | **2.20** |  | 99.96 |  | 100 |

BFE = Bacterial filtration efficiency (%)

**S25 - Sample calculation**

$$Bandana bacterial filtration efficiency$$

$$=\left[ 1-\frac{\left[ Droplets in the presence of test material \right]}{[Average positve control droplets (absence of test material)]} \right]\times100$$

$$Bandana bacterial filtration efficiency$$

$$=\left[ 1-\frac{\left[ 2309 \right]}{2361} \right]\times100$$

$$=2.2\%$$

1. Figure 7b,7c Data Set B: Control aerosol trials.

| d_50_ | N_R_ Positive control A | N_C_ Positive control A | N_R_ Positive control B | N_C_ Positive control B | N_R_ Negative control A | N_C_  Negative control A |
| --- | --- | --- | --- | --- | --- | --- |
| 7 | 178 | 236 | 170 | 221 | 0 | 0 |
| 4.7 | 214 | 306 | 233 | 349 | 0 | 0 |
| 3.3 | 296 | 539 | 269 | 447 | 0 | 0 |
| 2.1 | 325 | 670 | 309 | 592 | 0 | 0 |
| 1.1 | 287 | 506 | 260 | 420 | 0 | 0 |
| 0.65 | 9 | 9 | 14 | 14 | 0 | 0 |
| MPS |  | 3.02 |  | 3.12 |  | - |
| Total |  | 2266 |  | 2043 |  | 0 |
| MPS | 3.07 | | | |  |  |
| Total | 2155 | | | |  |  |

1. Figure 7b,7c Data Set B: Bacterial filtration efficiency trials.

| d_50_ | N_R_ Bandana | N_C_ Bandana | N_R_ 3-Ply Mask | N_C_ 3-Ply Mask | N_R_ N95 Respirator | N_C_ N95 Respirator |
| --- | --- | --- | --- | --- | --- | --- |
| 7 | 138 | 169 | 0 | 0 | 0 | 0 |
| 4.7 | 145 | 180 | 0 | 0 | 0 | 0 |
| 3.3 | 227 | 335 | 0 | 0 | 0 | 0 |
| 2.1 | 267 | 440 | 0 | 0 | 0 | 0 |
| 1.1 | 251 | 395 | 3 | 3 | 0 | 0 |
| 0.65 | 17 | 17 | 0 | 0 | 0 | 0 |
| MPS |  | 2.93 |  | 1.1 | - | - |
| Total |  | 1536 |  | 3 | 0 | 0 |
| BFE (%) |  | 28.71 |  | 99.86 |  | 100 |

1. Figure 7b,7c Data Set C: Control trials.

| d_50_ | N_R_ Positive control A | N_C_ Positive control A | N_R_ Positive control B | N_C_ Positive control B | N_R_ Negative control A | N_C_  Negative control A |
| --- | --- | --- | --- | --- | --- | --- |
| 7 | 220 | 319 | 186 | 250 | 0 | 0 |
| 4.7 | 245 | 379 | 206 | 289 | 0 | 0 |
| 3.3 | 324 | 664 | 289 | 513 | 0 | 0 |
| 2.1 | 353 | 857 | 335 | 727 | 0 | 0 |
| 1.1 | 343 | 779 | 328 | 686 | 0 | 0 |
| 0.65 | 28 | 29 | 27 | 28 | 0 | 0 |
| MPS |  | 2.93 |  | 2.85 |  | - |
| Total |  | 3027 |  | 2493 |  | 0 |
| MPS | 2.89 | | | |  |  |
| Total | 2760 | | | |  |  |

1. Figure 7b,7c Data Set C: Bacterial filtration efficiency trials.

| d_50_ | N_R_ Bandana | N_C_ Bandana | N_R_ 3-Ply Mask | N_C_ 3-Ply Mask | N_R_ N95 Respirator | N_C_ N95 Respirator |
| --- | --- | --- | --- | --- | --- | --- |
| 7 | 154 | 194 | 0 | 0 | 0 | 0 |
| 4.7 | 205 | 287 | 0 | 0 | 0 | 0 |
| 3.3 | 276 | 469 | 1 | 1 | 0 | 0 |
| 2.1 | 325 | 670 | 2 | 2 | 0 | 0 |
| 1.1 | 323 | 659 | 4 | 4 | 0 | 0 |
| 0.65 | 28 | 29 | 0 | 0 | 0 | 0 |
| MPS |  | 2.78 |  | 1.7 | - | - |
| Total |  | 2308 |  | 7 | 0 | 0 |
| BFE (%) |  | 16.38 |  | 99.75 |  | 100 |

BFE material testing was performed in accordance with ASTM F2101-19.^5^

**SECTION S10. STAGE DISTRIBUTION OF VIABLE BACTERIA-LADEN DROPLETS UNDER OPTIMIZED TEST CONDITIONS**

1. Stage distribution of viable bacteria-laden aerosol droplets

| Stage | Cut-off (µm) | Counts | SD | Trials |
| --- | --- | --- | --- | --- |
| 1 | ≥7 | 193 | 50 | 25 |
| 2 | 4.7 | 368 | 73 | 25 |
| 3 | 3.3 | 608 | 104 | 25 |
| 4 | 2.1 | 673 | 122 | 25 |
| 5 | 1.1 | 530 | 137 | 25 |
| **6** | **0.65** | **10** | **9** | **25** |

**Figure S3**. Stage distribution of viable bacteria-laden aerosol droplets (n=25).


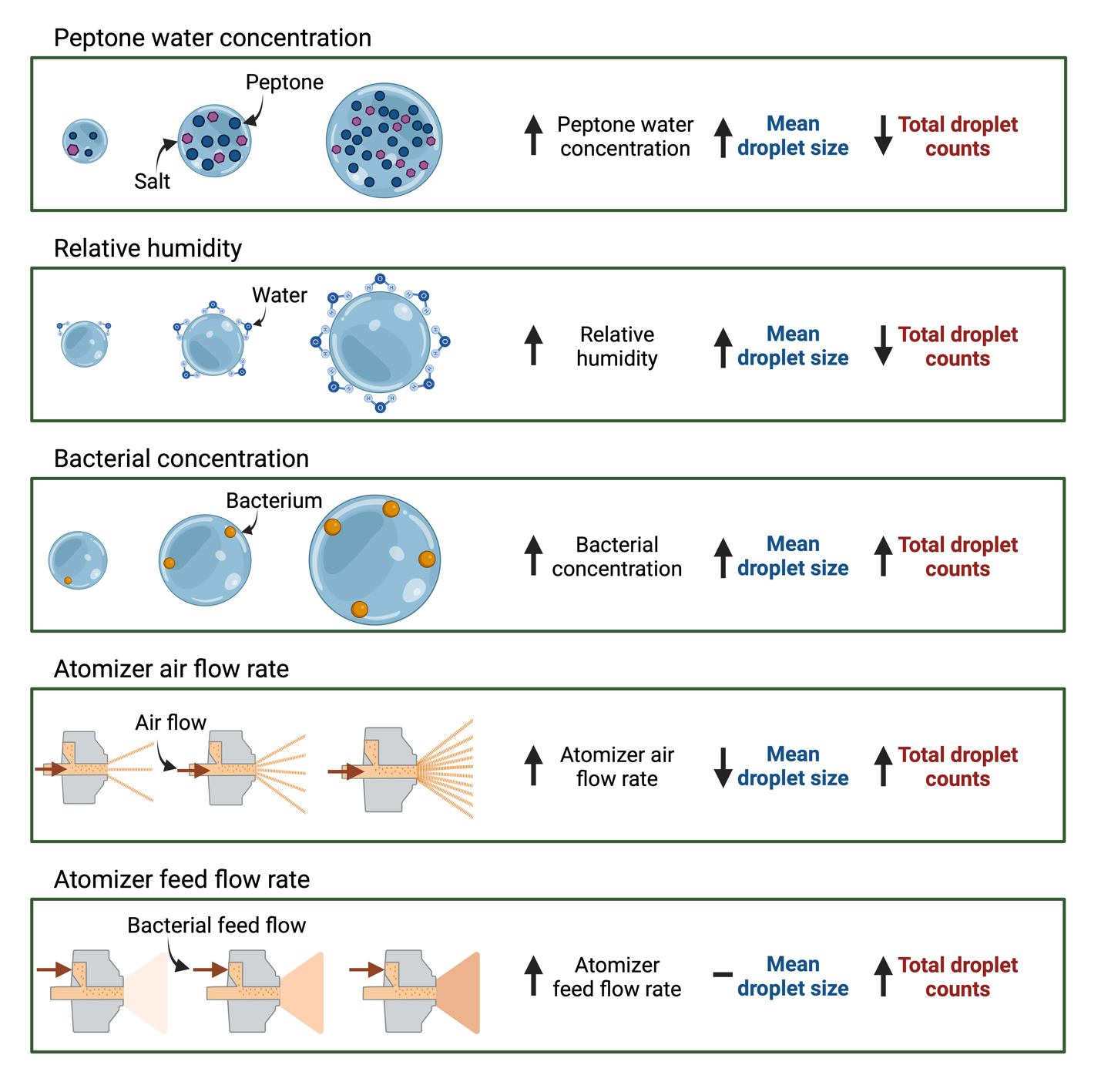
**Figure S4. The illustration depicts potential mechanisms impacting viable bacteria-laden droplet size and count.**

**REFERENCES**

1. ASTM F2101. Standard Test Method for Evaluating the Bacterial Filtration Efficiency (BFE) of Medical Face Mask Materials , Using a Biological Aerosol of Staphylococcus aureus. *American Society for Testing and Materials* **i**, (2019).

2. Mainelis, G. Bioaerosol sampling: Classical approaches, advances, and perspectives. *Aerosol Science and Technology* **54**, 496–519 (2020).

3. Crowder, T. M., Rosati, J. A., Schroeter, J. D., Hickey, A. J. & Martonen, T. B. Fundamental effects of particle morphology on lung delivery: Predictions of Stokes’ law and the particular relevance to dry powder inhaler formulation and development. *Pharmaceutical Research* vol. 19 Preprint at https://doi.org/10.1023/A:1014426530935 (2002).

4. Tishe Environmental. *Cascade Impactor Series 10-8XX Viable (Microbial) Particle Sizing Instruments Operations Manual*.

5. Macher, J. M. Positive-Hole Correction of Multiple-Jet Impactors for Collecting Viable Microorganisms. *Am Ind Hyg Assoc J* **50**, (1989).

6. Andersen, A. A. New sampler for the collection, sizing, and enumeration of viable airborne particles. *J Bacteriol* **76**, (1958).

7. F2100 Standard Specification for Performance of Materials Used in Medical Face Masks. https://www.astm.org/f2100-23.html.

8. BSI (British Standard Institute). BS EN 14683: Medical face masks. Requirements and test method. 26 (2019).
